# DELLA proteins are common components of symbiotic rhizobial and mycorrhizal signalling pathways

**DOI:** 10.1038/ncomms12433

**Published:** 2016-08-12

**Authors:** Yue Jin, Huan Liu, Dexian Luo, Nan Yu, Wentao Dong, Chao Wang, Xiaowei Zhang, Huiling Dai, Jun Yang, Ertao Wang

**Affiliations:** 1 National Key Laboratory of Plant Molecular Genetics, CAS Center for Excellence in Molecular Plant Sciences, Institute of Plant Physiology and Ecology, Shanghai Institutes for Biological Sciences, Chinese Academy of Sciences, Shanghai 200032, China; 2 University of Chinese Academy of Sciences, Beijing 100039, China; 3 Laboratory of Plant Biotechnology, College of Life and Environment Sciences, Shanghai Normal University, Shanghai 200234, China

## Abstract

Legumes form symbiotic associations with either nitrogen-fixing bacteria or arbuscular mycorrhizal fungi. Formation of these two symbioses is regulated by a common set of signalling components that act downstream of recognition of rhizobia or mycorrhizae by host plants. Central to these pathways is the calcium and calmodulin-dependent protein kinase (CCaMK)–IPD3 complex which initiates nodule organogenesis following calcium oscillations in the host nucleus. However, downstream signalling events are not fully understood. Here we show that *Medicago truncatula* DELLA proteins, which are the central regulators of gibberellic acid signalling, positively regulate rhizobial symbiosis. Rhizobia colonization is impaired in *della* mutants and we provide evidence that DELLAs can promote CCaMK–IPD3 complex formation and increase the phosphorylation state of IPD3. DELLAs can also interact with NSP2–NSP1 and enhance the expression of Nod-factor-inducible genes in protoplasts. We show that DELLA is able to bridge a protein complex containing IPD3 and NSP2. Our results suggest a transcriptional framework for regulation of root nodule symbiosis.

## Abstract (web summary)

Symbiotic associations between legumes and nitrogen-fixing bacteria are regulated by a CCaMK-IPD3 protein complex which promotes nodule initiation. Here, Jin *et al*. propose that DELLA proteins positively regulate nodulation by enhancing phosphorylation of IPD3 and acting as a positive regulator of transcription.

## Graphical abstract

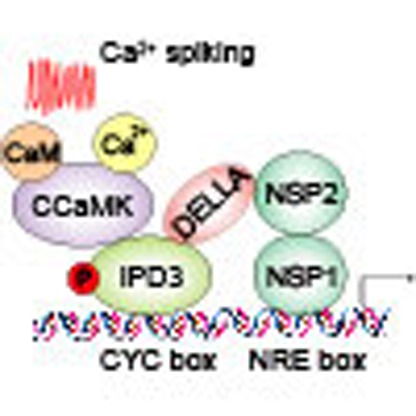

The availability of nitrogen is important for plant growth and stress resistance. Legumes are able to form beneficial symbiotic associations with nitrogen-fixing rhizobial bacteria that provide nitrogen to the host plant^1^,^2^. Legumes develop a specific organ, called a nodule, which provides the proper microenvironment for ammonia generation through the reduction of molecular dinitrogen by rhizobia and nutrient exchange between both symbionts. Nitrogen fixation is energetically expensive and this symbiotic interaction is precisely regulated by the plant to adapt to environment conditions.

There are two major developmental programs in root nodule symbiosis: nodule primordia originate from root cortical cells which re-activate cell divisions, and simultaneously, root epidermal responses prepare the cell for bacterial infection^2^. The formation of nitrogen-fixing nodules is controlled by a host genetic programme that synchronizes these two processes^3^,^4^. The early development of rhizobial symbiosis is achieved through a chemical communication between plant and rhizobium, present in the rhizosphere. Flavonoids are released by the plant roots as a signal to rhizobia. In turn, rhizobia produce nodulation factors (Nod factors) that are recognized by the host plant to activate a common symbiotic signalling pathway required for mycorrhizal and rhizobial symbioses^2^,^4^,^5^. Over the past 20 years, progress has been made in understanding the genetic programme in *Medicago truncatula* and *Lotus japonicus*. Two LysM receptor kinases NFR1/LYK3, NFR5/NFP and a leucine-rich repeat receptor kinase DMI2/SYMRK are necessary for Nod factor perception^6^,^7^,^8^,^9^,^10^,^11^,^12^. Interestingly, it has been recently shown that *NFR1*/*LYK3* is also involved in mycorrhizal symbiosis and is a new common symbiotic gene^13^. After perception at the plasma membrane, the remaining components are associated with nucleus, which are involved in both mycorrhizal and rhizobial symbioses. Two cation channels (Castor and Pollux) and three components at the core of the nuclear pore (NENA, NUP85 and NUP133) are located on the nuclear membrane^14^,^15^,^16^,^17^,^18^,^19^. These components are required for Nod-factor-induced calcium oscillations^4^. Perception of the calcium oscillations requires a nuclear-localized calcium and calmodulin-dependent protein kinase (CCaMK, known as DMI3 in *M. truncatula*)^20^,^21^. The activation of CCaMK is sufficient to induce symbiotic processes, as gain-of-function mutations in CCaMK induce nodulation in the absence of rhizobia^22^,^23^,^24^. CCaMK associates with and phosphorylates CYCLOPS (known as IPD3 in *M. truncatula*)^25^,^26^, which is essential for rhizobial and mycorrhizal colonization. Very recently, phosphorylated CYCLOPS has been shown to bind the promoter of *NIN* and induce nodulation in the absence of rhizobia^27^.

Several nuclear-associated transcriptional regulators, including nodule inception (NIN)^28^,^29^, an ERF family protein (ERN)^30^,^31^, and two GRAS family proteins, Nodulation signaling pathway 1 (NSP1) and NSP2, are required for the expression of Nod-factor-induced genes and initiation of nodulation^32^,^33^. NSP1 and NSP2 form a hetero-complex that associates with promoters of Nod-factor-inducible genes, such as *ENOD11* and *ERN1* (ref. 34)*. NSP1* and *NSP2* were originally thought to function specifically in nodulation. However, it was recently shown that *NSP1* and *NSP2* are also required for arbuscular mycorrhizal fungi (AMF)-associated lipochitooligosaccharide (LCO) signalling or mycorrhizal infection^35^,^36^,^37^,^38^. NSP1 contains a DNA-binding domain, whereas there is none in NSP2, which requires an interaction with NSP1 to associate with Nod-factor-inducible promoters^34^. The NSP1–NSP2 protein complex promotes the expression of *NIN* and *ERN*, two transcription factors with nodulation-specific functions. Interestingly, it has recently been shown that NSPs are not required for CYCLOPS-induced *NIN* expression, but they are required for CYCLOPLS-induced nodule organogenesis^27^. So it remains unknown how NSP1–NSP2 activity is associated with the action of CCaMK-CYCLOPS or whether the two protein complexes function independently in root nodule symbiosis.

The phytohormone gibberellic acid (GA) has been implicated in many aspects of plant biology, including seed germination, stem elongation, leaf expansion, pollen maturation and induction of flowering^39^,^40^. GA is perceived by GID1 (GA insensitive dwarf 1) and promotes the destruction of DELLA (SLR1, slender rice 1, one copy in rice genome) which is a nuclear protein that restrains the cell proliferation and expansion that drive plant growth^39^,^40^. DELLA is well known as a transcriptional suppressor that acts by directly binding DNA recognition domain of other transcript factors such as PIF3, 4 and inhibiting their transcriptional activities^41^,^42^. DELLA is an integrator of plant responses to hormones and environmental stresses, and DELLA-dependent growth restraints are advantageous in adverse environment stresses^43^.

DELLA family proteins are required for mycorrhizal symbiosis^44^,^45^. The mycorrhizal phenotype of *della* double mutant in *M. truncatula* shows DELLAs function at the arbuscule development stage^44^. Interestingly we recently showed that AMF infection and arbuscule development are both defective in the rice *della* mutant, *slr1*, suggesting an early signalling function of DELLAs in symbiosis^45^. This different mycorrhizal defect of mutants in rice and *Medicago* could be caused by genetic redundancy of DELLAs in *Medicago*. The role of GAs in the control of root nodule symbiosis has been investigated for over 60 years^46^,^47^. It has been shown that exogenous application of GAs inhibits the formation of infection threads and nodules, but other results show GAs can promote root nodule symbiosis in legumes^48^,^49^,^50^,^51^.

Here we show that DELLA proteins promote nodule development and infection thread formation during root nodule symbiosis. We provide evidence that DELLAs can promote CCaMK–IPD3/CYCLOPS complex formation and increase the phosphorylation of IPD3/CYCLOPS. We show that DELLAs can form a protein complex with NSP2–NSP1 and are able to bridge a protein complex containing IPD3/CYCLOPS and NSP2. We propose that DELLA proteins represent a missing link in the common symbiotic signalling pathway required for both rhizobial and mycorrhizal symbioses.

## Results

### GA inhibits root nodule symbiosis

**Figure 1:**
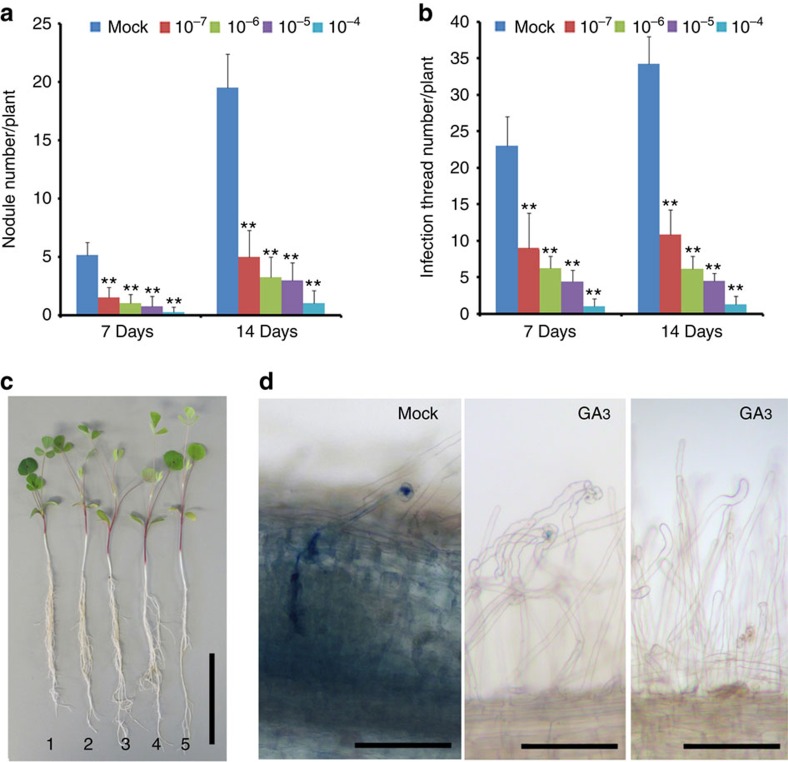
GA_3_ represses root nodulation development and rhizobial infection in *M. truncatula*. Wild-type A17 plants were grown with different concentrations of GA_3_ and upon inoculation with *S. meliloti* strain 1021 harbouring a *LacZ* reporter and assayed for nodule development (**a**), rhizobial infection events (**b**), and plant development (**c**). Increasing GA_3_ concentration promotes plant growth but inhibits nodulation and rhizobial infection. Infection threads were counted following *LacZ* staining. Twelve plants were analysed for each GA_3_ concentration. 1:mock, 2:10^−7^, 3:10^−6^, 4:10^−5^, 5:10^−4^ M GA_3_. (**d**) Representative images of infection threads visualized by staining of *S. meliloti* (strain 1021) expressing the *LacZ* reporter. The left panel shows infection thread progression in to a nodule primordium. The middle panel shows that a representative image of infection thread progression was inhibited by treatment of 10^−4^ M GA_3_ and the right panel shows that a representative image of no infection thread progression in the most plant roots treated with 10^−4^ M GA_3_. Scale bars correspond to 2 cm (**c**) and 100 μm (**d**). These experiments were repeated three times with similar results. Error bars are standard error. The asterisk indicates a significant decrease to the control with Student's *t*-test (**P<0.01).

It has previously been shown that gibberellins play an important role in root nodule symbiosis^48^,^49^,^50^. To better understand the nature of this regulation, we initiated studies to assess the effects of GA_3_ on nodulation efficiency in *M. truncatula* under our growth conditions. We grew *M. truncatula* cv. Jemalong line A17 plants in the soil pretreated with 0, 10^−7^, 10^−6^, 10^−5^ and 10^−4^ M GA_3_, concentrations of GA_3_ that are higher than endogenous GAs levels in plants, and quantified nodulation events at 7 and 14 days post inoculation with *Sinorhizobium meliloti* strain 1021 (*Sm1021*). As expected the shoots of GA-treated plants displayed increased shoot length and shoot fresh weight (Supplementary Fig. 1a,b). However, root length, lateral root number and root fresh weight of the plants treated with GA_3_ at concentrations from 10^−7^ to 10^−5^ M were not significantly changed compared with control plants (Supplementary Fig. 1c–e). Interestingly, we found that nodulation was severely impaired at 10^−6^–10^−4^ M GA_3_ treatment (Fig. 1a), while the shoot length increased after treated with GA_3_ (Fig. 1c). The generation of nodules involves a number of processes that may be regulated by GA_3_, including the re-initiation of cortical cell division to form a nodule primordium and the facilitation of bacterial infection through epidermal and cortical cells. We therefore assessed whether GA_3_ could regulate bacterial infection of the epidermis by quantifying the number of infection events and nodule development in plants grown at different concentrations of GA_3_. The infection frequency was also decreased with increasing concentrations of GA_3_ (Fig. 1b,d), suggesting that GAs regulates both nodule formation and bacterial infection independently of the shoot and root growth phenotype.

### DELLAs are required for root nodule symbiosis

**Figure 2:**
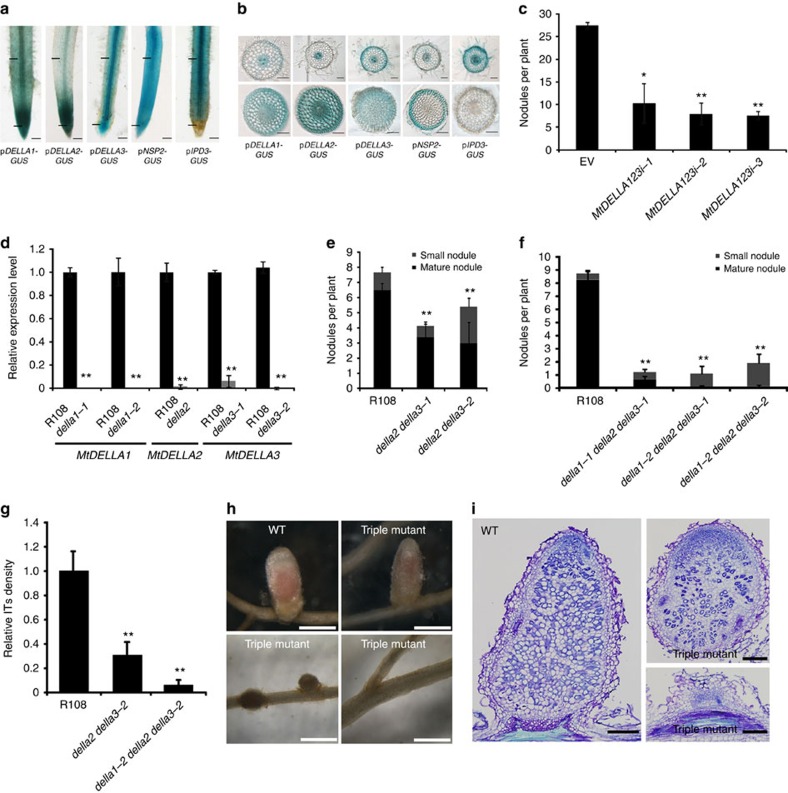
MtDELLAs promote root nodule symbiosis. (**a**) Whole-root GUS activity of p*DELLA1:GUS*, p*DELLA2:GUS*, p*DELLA3:GUS*, p*NSP2:GUS*, p*IPD3:GUS* are shown. Scale bars correspond to 2 mm. The black bars indicate that the region is selected for the transversal section in **b**. (**b**) Thin transversal sections (70 μm) of p*DELLA1:GUS*, p*DELLA2:GUS*, p*DELLA3:GUS*, p*NSP2:GUS*, p*IPD3:GUS* roots are shown. Scale bars correspond to 100 μm. (**c**) Numbers of nodules on *MtDELLAs* RNAi plant roots 4 weeks post inoculation with *S. meliloti*. *n*⩾8, where *n* denotes the number of plants. (**d**) qRT-PCR analysis of *MtDELLAs* transcript levels. The expression levels of *MtDELLAs* were detected in wild-type (R108) and *della1, della2* and *della3* mutants. Expression levels were normalized against the reference gene *Elongation factor 1-alpha* (*EF1-α*). The RNA was extracted from six individual plants of R108 or mutants. (**e**,**f**) Scores of nodule number in *della2*/*della3* double mutants (**e**) and *della1*/*della2*/*della3* triple mutants (**f**). (**g**) Quantification of infection threads in *della* double and triple mutants. The infection thread (IT) was visualized by staining of *S. meliloti* expressing the *LacZ* reporter and was counted at 7 d.p.i. *n*⩾13, where *n* denotes the number of plants. Relative ITs density indicates the number of infection threads per root length per plant, and they were normalized against the wild-type (R108). (**h**) Nodule development is impaired in *della1*/*della2*/*della3* triple mutant. There is no nodule formed in 12 plants out of 21 *della1-1/della2/della3-1* triple mutants. Occasionally pink nodules were formed in *della* triple mutants. Scale bars correspond to 500 μm. (**i**) Nodule sections of the pink nodule and the white nodule in the *della* triple mutant, nodules were stained with toluidine blue. Scale bars correspond to 200 μm. This is a representative experiment repeated three times in **c** and **d**, and twice in **e**, **f** and **g**. Error bars represent s.d. The asterisk indicates a significant decrease relative to wild type or vector control with Student's *t*-test (*P<0.05; **P<0.01).

Gibberellin is perceived by GID1 and promotes the interaction of GID1 and DELLA, which in turn leads to the polyubiquitination and subsequent degradation of DELLA by the 26s proteasome in the nucleus^39^,^40^. Three *DELLA* genes and two highly homologous genes were found in the *M. truncatula* genome database Mt3.5 and Mt4.0 (Supplementary Fig. 2)^44^. When we transformed functional *MtDELLA-GFP* (Supplementary Fig. 3) driven by 35S promoter into *M. truncatula* using hairy root transformation, no green fluorescent protein (GFP) signal was detected in the root, but occasionally very weak GFP signals were detected only in root tip cells, suggesting that the amounts of DELLA proteins are tightly regulated in *M. truncatula*. Consistent with the degradation of DELLAs by GA treatment in other plant species, GA_3_ treatment induced the degradation of MtDELLA2 and MtDELLA3 in the nucleus (Supplementary Fig. 3a). Under non-symbiotic conditions, the expression of the three *DELLAs* was detected in epidermal cells, cortical cells and stele in the rhizobial infection zone (closed to root apical meristem) and elongation zone (Fig. 2a,b). Consistent with our results, expression of *DELLA1* and *DELLA2* in the epidermal cells (root hair cells) was also found in transcriptional data (http://mtgea.noble.org/v3/blast_result.php?s=226645). Further analysis showed that *MtDELLA3* was upregulated 6 h post Nod factor treatment and that *MtDELLA1* and *MtDELLA2* were induced 7 day post inoculation (d.p.i.) with *Sm1021*, suggesting a role of DELLAs in root nodule symbiosis (Supplementary Fig. 4a,b). To assess whether *DELLAs* function in root nodule symbiosis, we generated transformed hairy roots expressing an *MtDELLA* hairpin allowing RNA-mediated interference of *MtDELLA*s. We used three different combination of RNA interference (RNAi) targeted three *MtDELLAs* and observed that expression levels of *MtDELLAs* were reduced in these RNAi plants (Supplementary Fig. 4e). The total nodule number was significantly decreased in *MtDELLA*s RNAi plants compared with the empty vector control (Fig. 2c), indicating that DELLA proteins are positive regulators in root nodule symbiosis.

To further confirm the RNAi results, *M. truncatula* lines containing *Tnt1* insertions in *MtDELLA1*, *MtDELLA2* and *MtDELLA3* were obtained from a mutant population generated at the Samuel Roberts Noble Foundation (Supplementary Fig. 4) (http://medicago-mutant.noble.org/mutant/database.php) and the transcription levels of *MtDELLA1*, *MtDELLA2* and *MtDELLA3* were greatly reduced in the *della* mutants (Fig. 2d). Given genetic redundancy of DELLAs in *Medicago*, *della2/della3* double mutants were generated and showed significantly decreased nodule number at 21 d.p.i. compared with its wild type (R108) (Fig. 2e). However, the root length of double mutant was not different from wild type (Supplementary Fig. 5). While few matured nodules were formed in the *della2/della3* double mutants (Fig. 2e). We further generated the *della* triple mutants and observed very few mature pink nodules in *della1/della2/della3* plants (Fig. 2f–i). Intriguingly, no nodules were formed in 12 out of 21 *della1-1/della2/della3-1* triple mutants. Further analysis revealed the number of infection threads was greatly reduced in *della* triple mutants and the pink nodule formed on *della* triple mutants appeared to be aberrant (Fig. 2f–i). These results indicated that *MtDELLAs* are essential for root nodule symbiosis.

### Gene induction by Nod factors is dependent on DELLAs

**Figure 3:**
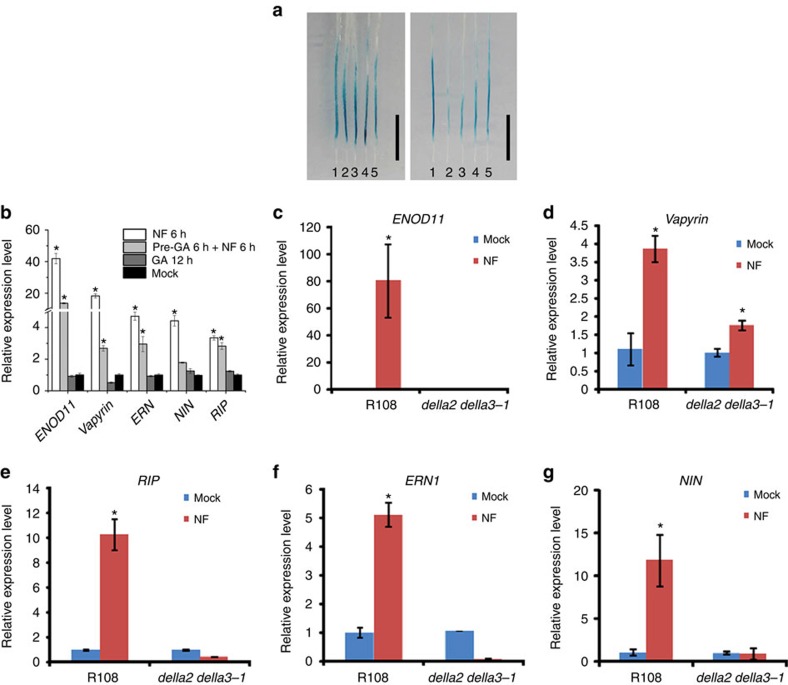
Nod-factor-induced gene expression is promoted by *MtDELLAs*. (**a**) The effect of GA_3_ on Nod-factor-induced gene expression was assayed using transgenic plants expressing p*ENOD11:GUS*. Note: 6 h pre-treatment of GA_3_ suppresses expression of *ENOD11* (right panel), but GA_3_ co-treatment with Nod factor does not suppress expression of *ENOD11* (left panel). 1:mock, 2:10^−4^, 3:10^−5^, 4:10^−6^, 5:10^−7^ M GA_3_. Scale bars correspond to 1 cm. (**b**) Real-time PCR results revealed that GA_3_ suppresses expression of Nod-factor-induced genes. Treatment with 1 nM Nod factor for 6 h induced expression of *ENOD11*, *VAPYRIN*, *NIN*, *ERN* and *RIP10* and the induction was reduced upon pre-treatment with 10^−5^ M GA_3_ for 6 h. (**c**–**g**) Real-time PCR results revealed that MtDELLAs required for induction of nodulin genes. Seven-day-old *della2/della3* double mutants and R108 were treated with 1 nM Nod factor or mock (BNM medium) for 6 h. In wild type the nodulin genes were induced, however these genes could not be induced in *della2/della3* double mutants after Nod factor treatment. These experiments were repeated three times with similar results, one representative experiment was shown. Error bars are standard error (*n*=3). The asterisk indicates a significant induction relative to control with Student's *t*-test (*P<0.01).

*ENOD11* represents one of the earliest marker genes for response to Nod factor and rhizobia. The induction of *ENOD11* requires the Nod factor signal transduction pathway and as such provides markers for the regulation of this signalling pathway^52^. To assess the effects of GA_3_ on Nod-factor-induced gene expression, we used *M. truncatula* plants stably transformed with β-glucuronidase (GUS) driven by the *ENOD11* promoter. Seven-day-old plants were transferred into liquid BNM medium containing 10^−7^, 10^−6^, 10^−5^ and 10^−4^ M GA_3_. Following 6 h of GA_3_ treatment, 1 nM Nod factor was added to the medium, and after 6 h of Nod factor treatment, the roots were used for GUS staining. Plants without Nod factor treatment only showed GUS activity in root caps, while Nod factor treatment activates *ENOD11* in a specific region of the root (Fig. 3a). Pre-treatment with 10^−4^ M GA_3_ led to reduced induction of *ENOD11* (Fig. 3a,b), but co-treatment with GA_3_ did not reduce induction of *ENOD11* (Fig. 3a,b), suggesting that DELLAs may act at a very early stage in the signal transduction pathway. Consistent with the GA treatment data, we found that the induction of Nod-factor-induced genes was impaired in *della2*/*della3* double mutants (Fig. 3c–g), suggesting that DELLAs may directly regulate the expression of early nodulin genes.

**Figure 4:**
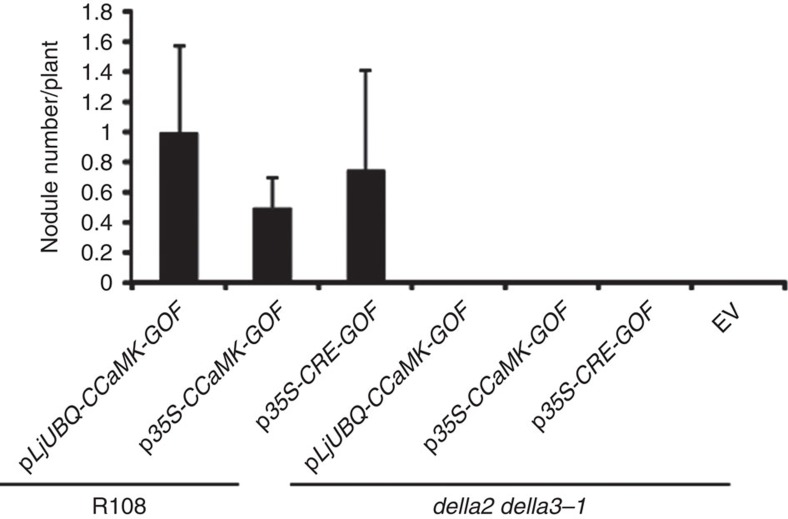
DELLAs promote spontaneous nodule formation induced by CRE1 and CCaMK. Spontaneous nodule formation induced by gain-of-function *CRE1* and *CCaMK1*-311 was blocked in *della* double mutants. The nodule number was counted 5 weeks after hairy root transformed plants being transferred to sterile vermiculite with perlite (*n*⩾6, where *n* denotes the number of plants).

A gain-of-function (GOF) mutation in a CCaMK or a gain-of-function of a cytokinin receptor (CRE1/SNF2) induces spontaneous cortical cells division and leading to spontaneous nodule formation in the absence of rhizobia^2^. The gain-of-function of *CCaMK* (1–311aa) or *GOF-CRE1* was introduced into hairy roots to determine the effect of GA_3_-coupled DELLA degradation on the cortical cell division. The transgenic roots treated with GA_3_ showed a significant reduction in the number of spontaneous nodules in *GOF-CRE1* lines (Supplementary Fig. 6a), suggesting that DELLAs are required for spontaneous nodule formation induced by cytokinin and DELLAs may function at the nodule development stage. Spontaneous nodule formation was also inhibited by GA_3_ treatment in overexpression of *GOF-CCaMK* plants (Supplementary Fig. 6b). Intriguingly, the spontaneous nodule formation induced by *CRE1-GOF* or *CCaMK1-311-GOF* was fully blocked in *della2*/*della3-1* double mutants (Fig. 4), suggesting that DELLA proteins act downstream of *CCaMK*.

### DELLAs are able to interact with IPD3

**Figure 5:**
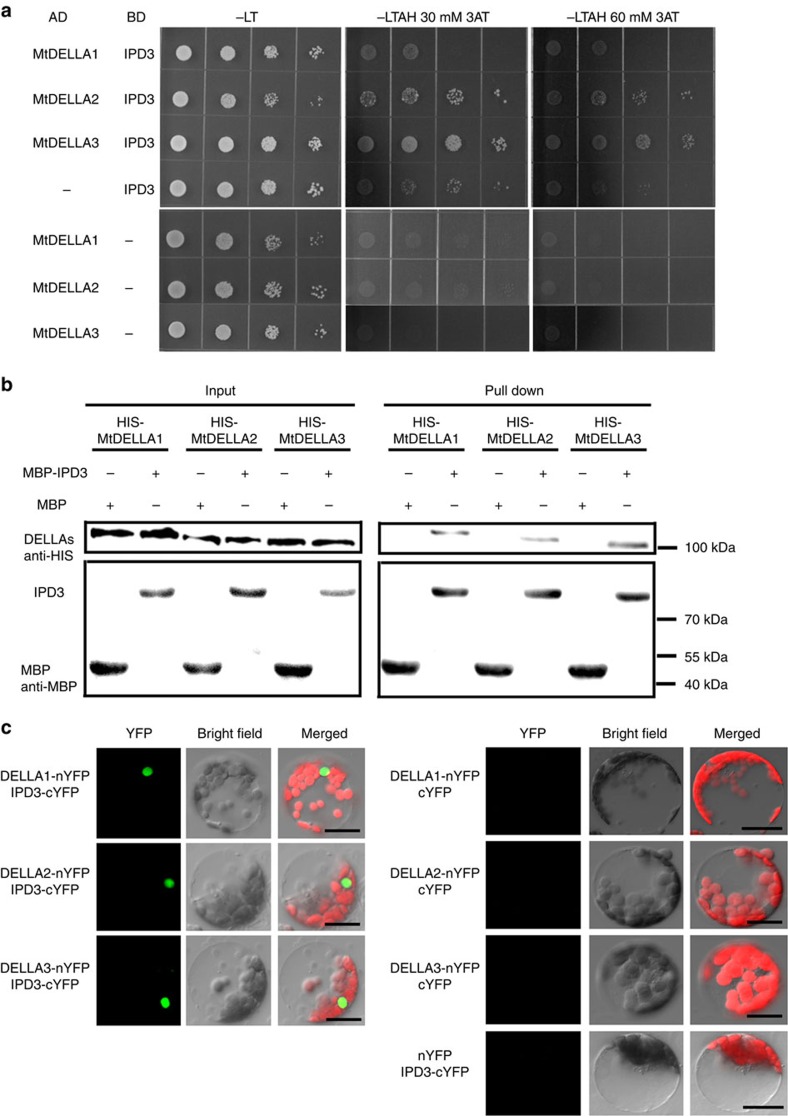
Interactions between MtDELLAs and MtCYCLOPS/IPD3. (**a**) Yeast two-hybrid assays between IPD3 and MtDELLA1, MtDELLA2 or MtDELLA3. Yeast cells carrying different combinatory constructs are listed on the left. Serial dilutions (10 times) of yeast cells expressing the indicated proteins from the pGADT7 (AD) and pGBKT7 (BD) vectors were plated onto SD/-Leu-Trp (-LT) medium or SD/-Leu-Trp-Ade-His (-LTAH) medium with 30 or 60 mM 3-amino-1,2,4-triazole (3AT). (**b**) Pull-down assays between IPD3 and MtDELLA1, MtDELLA2 or MtDELLA3. MBP-IPD3 fusion protein but not MBP alone bound with HIS-tagged MtDELLA1, MtDELLA2 or MtDELLA3. (**c**) Detection of protein–protein interactions in *Arabidopsis* protoplast by BiFC. YFP fluorescence of leaves co-transformed with MtDELLAs-nYFP (N-terminal half of YFP) and IPD3-cYFP (C-terminal half of YFP). No protein interactions were detected in the other three combinations: MtDELLAs-nYFP--cYFP, IPD3-cYFP--nYFP and cYFP--nYFP. Scale bars, 20 μm.

Since DELLA proteins are involved in both root nodule and mycorrhizal symbioses, we propose that DELLAs may interact with the key components of the common signalling pathway. We observed that DELLA proteins MtDELLA1, MtDELLA2 and MtDELLA3 interacted with IPD3, but not CCaMK and NSP1 in a yeast two-hybrid (Y2H) assay, although CCaMK and NSP1 proteins were expressed in yeast (Fig. 5a; Supplementary Fig. 7c,d). MtDELLAs and IPD3 are also able to interact in *in vitro* pull-down assays (Fig. 5b) and bimolecular fluorescence complementation (BiFC) assays in *Arabidopsis* protoplasts (Fig. 5c). A strong fluorescence signal was observed in the nucleus of *Arabidopsis* mesophyll protoplasts co-transformed with IPD3-nYFP and MtDELLAs-cYFP. Protoplasts transformed with *MtDELLA-nYFP* and the control vector or with *IPD3-cYFP* and the control vector were used as controls, which showed no positive signal (Fig. 5c). Taken together, these results show that MtDELLAs are able to interact with IPD3.

### DELLAs can form a protein complex with IPD3 and CCaMK

**Figure 6:**
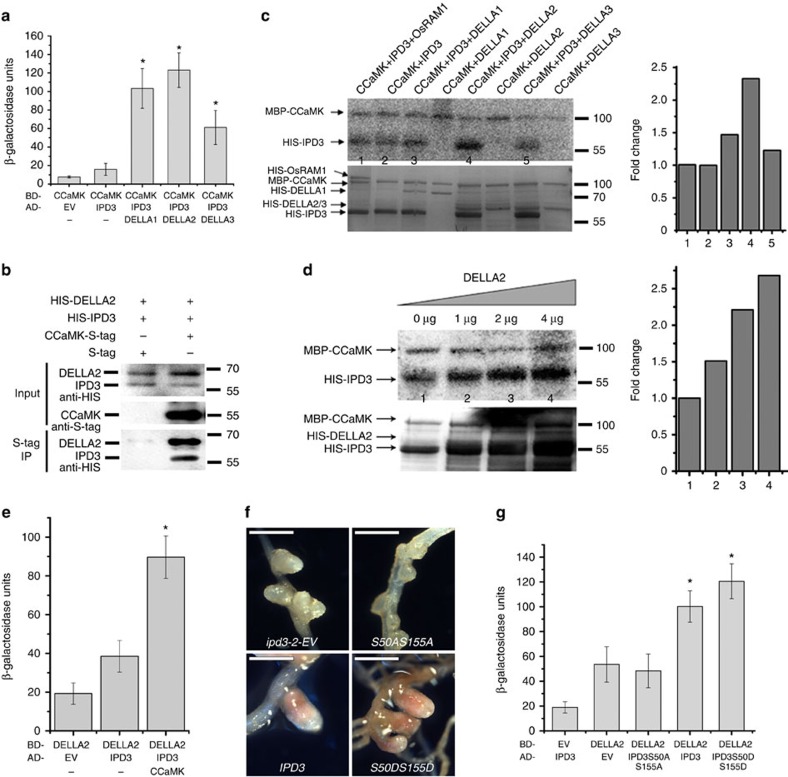
CCaMK–IPD3–DELLA protein complex formation. (**a**) MtDELLAs increased the interaction between CCaMK and IPD3 in yeast three-hybrid assays. Data are presented as β-galactosidase activity. (**b**) CCaMK co-immunoprecipitates with IPD3 and DELLA2 in *E. coli*. CCaMK, IPD3 and MtDELLA2 are co-expressed in *E. coli*. (**c**) The effect of MtDELLAs on phosphorylation of IPD3 by CCaMK *in vitro* phosphorylation assays. The left histogram shows quantitative intensities of IPD3 phosphorylation calculated by ImageJ. The relative intensities were normalized against the control (bar1). (**d**) MtDELLA2 increased the phosphorylation of IPD3 by CCaMK. An increasing amount of MtDELLA2 was added to the reaction mix. The left histogram shows quantitative intensities of IPD3 phosphorylation calculated by ImageJ. The relative intensities were normalized against the bar1. Autoradiographs show the corresponding phosphorylated IPD3. Autoradiographs of kinase assays (^32^P) (upper images) and Coomassie staining of the gels (lower image) in **c** and **d**. This is a representative experiment that was repeated twice in **c** and **d**. (**e**) CCaMK increased interaction of MtDELLA and IPD3 in yeast three-hybrid assay. Data are presented as β-galactosidase activity. (**f**) *M. truncatula ipd3-2* roots were transformed with *IPD3-S50A-S155A*, *IPD3-S50D-S155D* and *IPD3*. The pink nodules were formed on *ipd3-2* mutants contained *IPD3* and *IPD3-S50D-S155D* upon 35 day post inoculation with *Sm1021*. Scale bars correspond to 500 μm. (**g**) Yeast three-hybrid assay showed sites S50 and S155 of IPD3 are critical for interaction of IPD3 and MtDELLA. Alanine replacement of S50 and S155 of IPD3 abolished the interaction with MtDELLA. Data are presented as β-galactosidase activity. Results represent the means of three experiments in **a**, **e** and **g**. Error bars represent standard error. The asterisk indicates a significant increase relative to the control with Student's *t*-test in **a**, **e** and **g** (*P<0.01).

It has been shown that CCaMK interacts with and phosphorylates IPD3/CYCLOPS^26^. To assess the relationship between CCaMK, IPD3 and MtDELLAs, we performed a yeast three-hybrid (Y3H) assay with the fusion proteins CCaMK-GAL4-DNA-binding domain (GAL4-DBD) and/or IPD3-GAL4 activation domain (GAL4-AD). Additive activation of the reporter was observed when MtDELLA proteins were present (Fig. 6a), suggesting that MtDELLA may form a complex with CCaMK and IPD3. We did not observe the interaction of CCaMK and MtDELLAs in the yeast two-hybrid assay (Supplementary Fig. 7). To further assess whether MtDELLA, CCaMK and IPD3 can form a protein complex, we co-expressed HIS-DELLA, CCaMK-S-tag and HIS-IPD3 in *Escherichia coli* and found that CCaMK could co-immunoprecipitate both IPD3 and DELLA2 (Fig. 6b), suggesting that MtDELLA, CCaMK and IPD3 are able to form a complex.

### DELLA enhances IPD3 phosphorylation

To analyse whether CCaMK could phosphorylate MtDELLAs, *in vitro* kinase assays were performed (Fig. 6c,d). We found that CCaMK could not directly phosphorylate MtDELLAs, but addition of MtDELLAs was able to enhance the intensity of phosphorylation of IPD3 by CCaMK (Fig. 6c,d). These results suggest that MtDELLAs may participate in the nodulation signalling pathway by interacting with IPD3 and promoting its phosphorylation. Interestingly we found that CCaMK could further increase the interaction of MtDELLA and IPD3 in a Y3H assay (Fig. 6e), suggesting that calcium could be an important signal for the formation of CCaMK–IPD3–DELLA complex during root nodule symbiosis. The two phosphorylated serine residues within the N-terminal of CYCLOPS/IPD3 are critical for symbiosis in *Lotus japonicas*^27^. In *M. truncatula*, we also found that the two residues IPD3 S50-S155 are essential for symbiosis (Fig. 6f). The phosphoablative mutant version of IPD3 S50A-S155A could not complement the *ipd3-2* mutant while the phosphomimetic version of S50D-S155D could fully complement *ipd3-2* (Fig. 6f). Interestingly we found that phospoablative mutant version of S50A-S155A also abolished the interaction with MtDELLAs (Fig. 6g; Supplementary Fig. 7). While the precise reason for the phenotype of the phosphoablative mutant remains to be determined, the inability of phospoablative mutant version to interact with MtDELLA, suggests that this interaction may be relevant for symbiosis. Based on these results we propose that phosphorylation of IPD3 by CCaMK promotes assembly of a CCaMK–IPD3–DELLA complex during root nodule symbiosis.

### DELLAs–NSP2–NSP1 activates Nod-factor-induced genes

**Figure 7:**
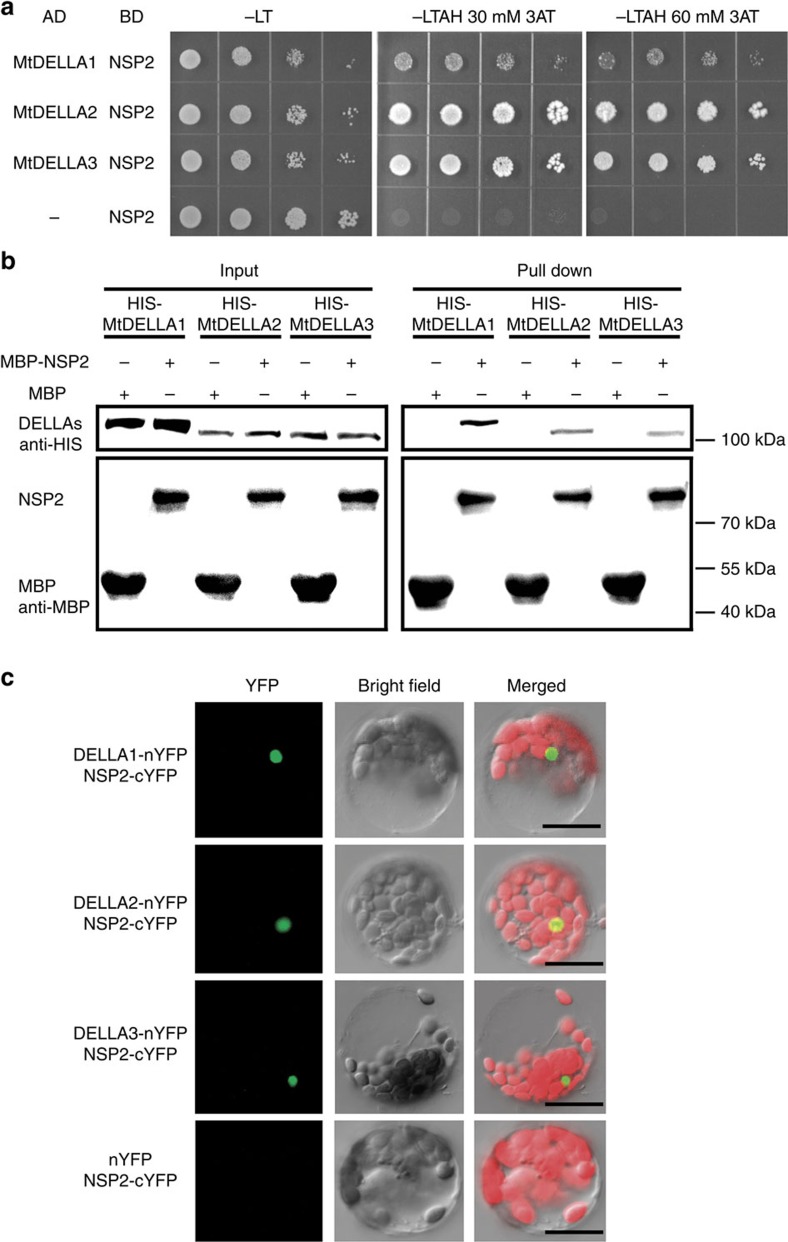
Interactions between MtDELLAs and NSP2. (**a**) Yeast two-hybrid assays between NSP2 and MtDELLA1, MtDELLA2 or MtDELLA3. Yeast cells carrying different combinatory constructs are listed on the left. Serial dilutions (10 times) of yeast cells expressing the indicated proteins from the pDEST-GADT7(AD) and pDEST-GBKT7(BD) vectors were plated onto SD/-Leu-Trp(-LT) medium or SD/-Leu-Trp-Ade-His (-LTAH) medium with 30 or 60 mM 3-amino-1,2,4-triazole (3AT). (**b**) Pull-down assays between IPD3 and MtDELLA1, MtDELLA2 or MtDELLA3. MBP-IPD3 fusion protein but not MBP alone bound HIS-tagged MtDELLA1, MtDELLA2 or MtDELLA3. (**c**) Detection of protein–protein interactions in *Arabidopsis* protoplast by BiFC. YFP fluorescence of leaves co-transformed with MtDELLAs-nYFP and NSP2-cYFP. There is no protein interaction in the other three combinations: MtDELLAs-nYFP--cYFP, NSP2-cYFP--nYFP and cYFP--nYFP (Fig. 5c). Scale bars, 20 μm.

**Figure 8:**
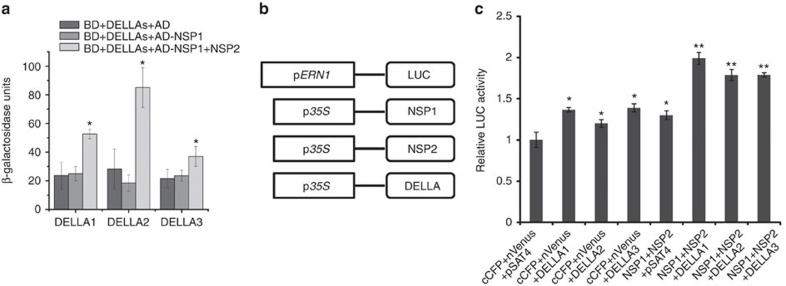
The effect of DELLAs on NSP2–NSP1 induced expression of *ERN1*. (**a**) Yeast three-hybrid assay assessing combinations of BD-MtDELLAs, AD-NSP1 and NSP2, showing MtDELLAs form a protein complex with NSP1 and NSP2. Data are presented as β-galactosidase activity. The asterisk indicates a significant increase relative to the control with Student's *t*-test (*P<0.05). (**b**,**c**) A transient reporter assay was used to test the trans-activation effects of NSP1, NSP2 and MtDELLAs on the expression of *ERN1* in *Arabidopsis* protoplasts. (**b**) Schematic represent of the effectors and the reporter. (**c**) The activity caused by vector control, NSP1–NSP2 and MtDELLAs. The asterisk (*) indicates a significant increase relative to empty vector control (*P*<0.05), the asterisk (**) indicates a significant increase relative to empty vector control and NSP1–NSP2 (*P*<0.01). Results represent the means of three experiments. Error bars represent standard error.

It was recently shown that *NSP2* is also required for AMF-associated LCO signalling^36^,^53^. We therefore assessed whether MtDELLAs can interact with NSP2, which is a GRAS family member that directly binds to a specific promoter region of Nod-factor-inducible genes through interaction with NSP1. We found that NSP2 could interact with MtDELLA proteins DELLA1, DELLA2 and DELLA3 in Y2H (Fig. 7a), pull-down and BiFC assays (Fig. 7b,c). Intriguingly, we further observed a DELLAs–NSP2–NSP1 protein complex using the Y3H assay (Fig. 8a). It has been shown NSP1 and NSP2 could form complex and activate the expression of *ERN1*^34^. As DELLA proteins function as positive regulators of root nodule symbiosis, we hypothesized that MtDELLAs could function as transcriptional activators to regulate gene expression. Transient activation analysis in *Arabidopsis* protoplasts showed that transfection of the protoplasts with *NSP1* and *NSP2* could induce a low expression level of p*ERN1-LUC* (luciferase reporter gene driven by the *ERN1* promoter) compared with the empty vector control (Fig. 8b,c), which is consistent with a previous report^54^. Co-transfection of *NSP1*/*NSP2* with one of the *MtDELLAs* led to a much higher additive induction of p*ERN1-LUC* (Fig. 8b,c). These results suggest that MtDELLAs can function as transcription activators and regulate the expression of Nod-factor-induced genes with NSP1/NSP2 in protoplasts.

### DELLA can bridge a protein complex containing IPD3 and NSP2

**Figure 9:**
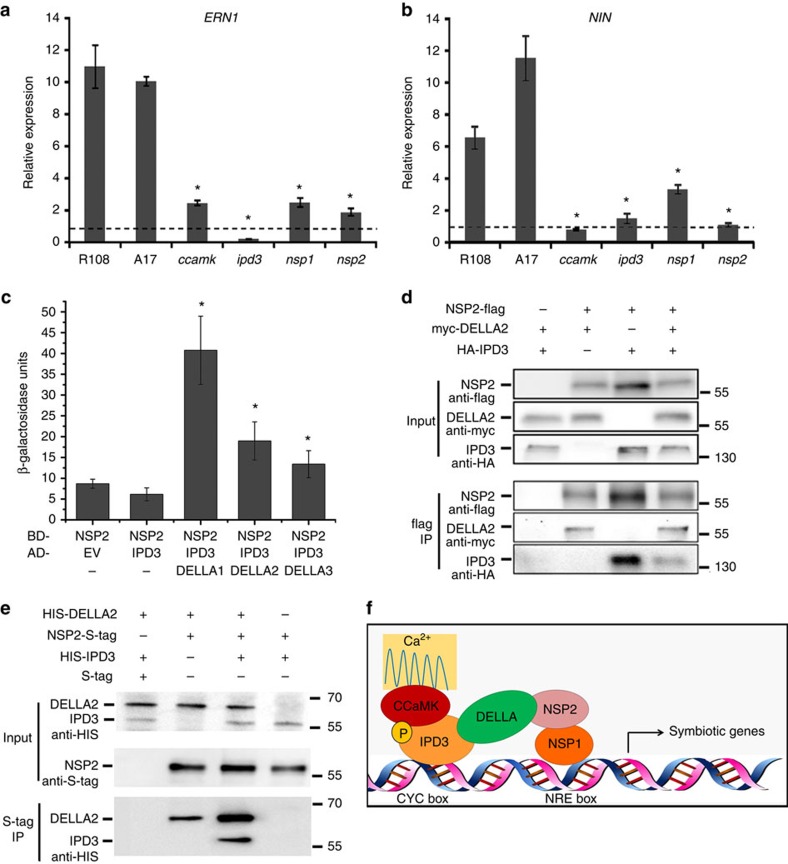
MtDELLAs can link IPD3 and NSP2. (**a**,**b**) The *dmi3-1*, *ipd3-2*, *nsp1-2* and *nsp2-1* mutants show defect in *NIN* and *ERN1* expression. The RNA was extracted from 6 individual plants of wild type or mutants. Error bars represent standard error (*n*=3, where n denotes the number of technical repetition). The asterisk indicates a significant decrease relative to the control with Student's *t*-test (* *P*<0.05) (**c**) Yeast three-hybrid assay assessing combinations of BD-NSP2, AD-IPD3 and MtDELLAs, indicating MtDELLA proteins bridge a protein complex containing IPD3 and NSP2. Data are presented as β-galactosidase activity. The asterisk indicates a significant increase relative to the control with Student's *t*-test (*P<0.05) (**d**) NSP2 co-immunoprecipitates with IPD3 and DELLA2 in *N. benthamiana*. NSP2, IPD3 and MtDELLA2 are co-expressed in *N. benthamiana*. (**e**) NSP2 co-immunoprecipitates with IPD3 only in the presence of MtDELLA. NSP2, IPD3 and MtDELLA are co-expressed in *E. coli*. (**f**) Proposed model showing MtDELLAs bridge a bigger protein complex containing CCaMK–IPD3 and NSP2–NSP1.

CCaMK–IPD3 and NSP2–NSP1 can both independently regulate the expression of Nod-factor-induced genes in *Nicotiana benthamiana*, suggesting that the two protein complexes may function independently^27^,^34^,^55^. However, the fact that we found that expression of both *NIN* and *ERN1* was lower in *ccamk*, *ipd3*, *nsp1* and *nsp2* mutants (Fig. 9a,b) suggests that the CCaMK–IPD3 and NSP2–NSP1 complexes may both regulate the expression of common Nod-factor-induced genes. We assessed whether MtDELLAs may be able to link IPD3 and NSP2 using Y3H in combination with the fusion proteins NSP2-GAL4-DNA-binding domain (GAL4-DBD) and/or IPD3-GAL4 activation domain (GAL4-AD). Activation of the reporter was observed only when MtDELLA protein was present, suggesting that MtDELLA bridges an interaction between IPD3 and NSP2 (Fig. 9c). A protein complex containing MtDELLA, IPD3 and NSP2 was also suggested by co-immunoprecipitation in *N. benthamiana* leaves (Fig. 9d). Interestingly, we found that IPD3 can co-immunoprecipitate with NSP2 in the absence of MtDELLA in *N. benthamiana*, possibly because the endogenous NbDELLA or another protein may also be able to form a protein complex with NSP2 and IPD3. To further test whether MtDELLA is able to bridge a protein complex containing IPD3 and NSP2, we co-expressed NSP2-S-tag and HIS-IPD3 in *E. coli* and found that NSP2 could not co-immunoprecipitate with IPD3, however NSP2 can co-immunoprecipitate with IPD3 in the presence of MtDELLA (Fig. 9e). Taken together, our results suggest that MtDELLA may be able to bridge a protein complex containing IPD3 and NSP2 (Fig. 9f).

## Discussion

Previous studies have shown that GA biosynthetic genes were upregulated during arbuscule mycorrhizal symbiosis and both positive and negative roles of GAs in mycorrhizal symbiosis have been suggested^56^,^57^,^58^,^59^,^60^. The role of GAs in legume root nodule symbiosis has been assayed for over 60 years^46^,^47^. The upregulation of GA biosynthetic genes at different stages of nodule development suggests an important role for GA in early infection and the mature stages of nodulation, such as nodule primordium development and the correct establishment of nodulation zones^49^,^50^,^51^. The *Pisum sativum* GA biosynthetic mutant, *na-1*, develops significantly fewer and underdeveloped nodules than its wild-type parent and GA application and grafting suggested that this nodulation phenotype in the *na-1* mutant is due to reduced GA level in the roots^51^, suggesting that GAs play a positive role in cortical cell divisions or nodule maturation. Here we found that DELLA is a positive regulator at the infection and the root nodule development stages. Taken together, these results suggest that strict regulation of DELLA proteins levels is required for normal development of mycorrhizal and root nodule symbioses, but the underlying mechanism needs to be further understood.

Legume plants share a common symbiotic signalling pathway to perceive LCO signalling molecules from rhizobial bacteria and mycorrhizal fungi, and activate the symbiotic signalling pathway^4^,^61^. It has been shown previously that DELLAs are essential for mycorrhizal symbiosis in rice and *M. truncatula*^44^,^45^. Here we show that *MtDELLAs* are also required for root nodule symbiosis and therefore represent a new common component of the signalling pathways required for root nodule and mycorrhizal symbioses. There are five DELLA proteins in *Arabidopsis* and only one DELLA protein in rice, suggesting that the DELLA family of proteins may have undergone several duplications during the evolution of *Arabidopsis*. Based on the available genome information for *M. truncatula*, we found three proteins containing DELLA domains and two GRAS proteins which fall into the cluster with DELLA proteins. We found that GA_3_ treatment severely inhibited rhizobial infection and root nodule development programmes. Consistent with this, we found that the nodule number was further reduced in *della* triple mutants. A total of 14% nodules still formed in triple mutants compared with wild type possibly because unknown *MtDELLAs* or other highly homologous GRAS proteins may also function during root nodule development and compensate for the mutation of the *MtDELLAs* we describe here.

Here we showed that MtDELLAs can increase the interaction between CCaMK and IPD3/CYCLOPS and lead to a higher level phosphorylation of IPD3 by CCaMK, indicating that MtDELLAs may be involved in assembly of a functional CCaMK-IPD3-DELLA protein complex. It has been shown that phosphorylated versions of CYCLOPS/IPD3 can bind the promoter of *NIN* and activate *NIN* expression^27^. Interestingly we found that a phospoablative mutant version of IPD3 (S50A-S155A) abolished the interaction with MtDELLAs and was unable to rescue an *ipd3* mutant. A phosphomimetic version (S50D-S155D) increased the interaction with DELLAs. These results are consistent with a model where the interaction of MtDELLAs and IPD3 is relevant for symbiosis. Furthermore we found CCaMK could further increase the interaction of MtDELLA and IPD3, suggesting that assembly of the CCaMK–IPD3–DELLA complex could be activated by CCaMK during root nodule symbiosis.

In vertebrates, CaMKIV contributes to phosphorylation of CREB on S133. Once phosphorylated on S133, CREB recruits its co-activator CBP, then CaMKIV phosphorylates CBP and thereby stimulates CREB-dependent transcription^62^. Our data suggests that the CCaMK/IPD3/DELLA complex could act via a similar mechanism in root nodule symbiosis as CaMKIV/CREB/CBP. Our data suggests that CCaMK can phosphorylate IPD3/CYCLOPS and that the phosphorylated version of IPD3 can interact with MtDELLAs. We found that DELLA can also increase the interaction between CCaMK and IPD3/CYCLOPS and stimulate higher levels of IPD3/CYCLOPS phosphorylation. However, we did not observe CCaMK phosphorylation of MtDELLAs *in vitro*, which is different from the calcium decoding system in vertebrates.

DELLAs are key negative regulators of gibberellin signalling^40^. It has been shown that DELLA interacts with other transcription factors, such as PIF3 and PIF4 to block their DNA-binding abilities of targeting gene promoters and inhibit their transcriptional activities^41^,^42^. Here we show that MtDELLAs are required for the expression of Nod-factor-induced genes, indicating MtDELLAs function as positive regulators in this context. Consistent with their role in root nodule symbiosis, we found that MtDELLAs can interact with another GRAS transcription factor, NSP2. Furthermore, MtDELLA, NSP2 and NSP1 were able to form a protein complex (Fig. 9f). Transient assays in *Arabidopsis* protoplasts showed that a luciferase reporter controlled by the *ERN1* promoter was additively transactivated by MtDELLAs, suggesting that MtDELLAs may function as transcriptional activators during root nodule symbiosis.

The binding sites for the CYCLOPS/IPD3 proteins (CYC-box) and NSP1 (NRE-box) in the *NIN* promoter are separated by gaps of 8 and 54 bps (Supplementary Note 1). This close proximity in DNA-binding sites suggest an interaction between the NSP2–NSP1 and CCaMK–IPD3 protein complexes may be possible. Consistent with this idea, we found that MtDELLAs are able to bridge a protein complex containing IPD3 and NSP2. The NRE-box and CYC-box may not always be associated in promoters of Nod-factor-induced genes, suggesting that NSP2–NSP1 and CCaMK–IPD3 many also function independently. We propose that NSP2–NSP1, CCaMK–IPD3 and other transcription factors may act in combination to regulate the expression of early nodulin genes with appropriate spatial and temporal patterns, and based on phenotype, that the MtDELLAs act during the early stages of signal transduction.

The legume–rhizobia symbiosis is the most important symbiotic association in terms of biological nitrogen fixation^63^,^64^, however nitrogen fixation is energetically expensive to the plant, so a number of additional external and internal factors negatively regulate nodulation in legumes^64^. Here, we show that the transcriptional repressor MtDELLA proteins, which are central regulators of GA signalling promote nodulation in *M. truncatula* and may act as crosstalk nodes to integrate hormone signals with rhizobial infection and nodule development.

## Methods

### Rhizobia inoculation and GA_3_ treatment

*M. truncatula* seeds were treated with 98% sulfuric acid and plated onto 1% agar medium at 4 °C. After about 3 days, they were moved to 22 °C overnight for germination. Then the seedlings were moved to a mixed soil containing 1:1 ratio of sand and perlite. After a minimum of 3 days, plants were inoculated with *S. meliloti*. Seeds of R108 or A17 were used as the wild type. Plants were grown in a greenhouse at 22 °C with 16/8 h of light/dark cycle at 22 °C. *S. meliloti* strain *Sm1021* was incubated in liquid Luria broth overnight with 400 μg ml^−1^ streptomycin selection. Bacteria were pelleted at 2,000*g* for 15 min and resuspended in H_2_O to OD_600_=0.03.

A concentration of 10 mM GA_3_ stock solution (GA_3_; G-7645-1G; Sigma) was dissolved using ethanol and diluted with ultra-pure H_2_O to a final working solution of 10^−4^ or 10^−5^ M GA_3_. Ethanol (0.1%) was used as mock solution. Three days after germination, 1 ml GA_3_ working solution or mock solution was applied daily to each plant of WT or mutants.

*MtDELLAs* RNAi *M. truncatula* plants were produced by *Agrobacterium rhizogenes* (Arqual) mediated hairy root transformation^65^. The candidate sequences that can be targeted by RNAi were selected according to clontech RNAi Target Sequence Selector (http://bioinfo.clontech.com/rnaidesigner/sirnaSequenceDesign.do;jsessionid=c0a8fe1e501a50bb6ab39948a9982afb46cf4fb4dd) and were amplified by PCR (primers seen in Supplementary Table 1) from wild-type gDNA. Fragments were cloned to the vector pK7GWIWGII-R, which carry a dsRed fluorescent tag for selection of transgenic hairy roots. The plants were transferred to sand with perlite about 1 month after transformation, and nodules were scored 3 weeks post inoculation with *Sm1021*.

### Genotyping of *della* mutants

We obtained *M. truncatula* R108 *Tnt1* transposon insertion lines of *Mtdella1-1* (NF4215), *Mtdella1-2*(NF5155), *Mtdella2* (NF14614), *Mtdella3-1* (NF11992) and *Mtdella3-2* (NF15025) from the Noble Foundation *Tnt1* database (http://medicago-mutant.noble.org/mutant/database.php). The location of *Tnt1* insertions in *MtDELLAs* were shown in Supplementary Fig. 4. These mutants were grown and genotyped by PCR using Tnt1-R1 and *DELLAs* gene-specific primers (Supplementary Table 1). NF14614 was crossed with NF11992 or NF15025 to generate double mutants. Then *della2/della3* double mutants were crossed with NF5155 or NF4215 and *della1/della2/della3* triple mutants were segregated from population of *della* triple heterozygous mutants.

### Vector information

For yeast two-hybrid constructs, the appropriate genes were cloned into the Gateway donor vector pEntry-topo-SD (appropriate primers see Supplementary Table 1), sequenced and then recombined into the pGBKT7GW and pGADT7GW vectors by LR reactions (Invitrogen). For yeast three-hybrid constructs, pBridge was employed and all the genes were introduced into MCSI and MCSII with ligation high (TOYOBO) except *DELLA3*, which was recombined to the MCSII by ClonExpressTM II One Step Cloning Kit (Vazyme). For the BiFC vectors, the genes of interest were cloned into the pSAT4-nYFP-N1 and pSAT4-cYFP-N1 with appropriate restriction enzymes cleavage sites (Supplementary Table 1). For protein expression in *E. coli*, pET28a, pET32a, and pMal-C2X were used (for primers see Supplementary Table 1). For transient reporter assays, the 2.4 kb *ERN1* promoter region was amplified by PCR from *M. truncatula* R108 genomic DNA and cloned by replacing the 2 × 35S promoter in the pSAT1-cCFP-C (pE3242), a protoplast expression vector. The firefly luciferase gene was amplified by PCR from pH35S-LUC-GW plasmid (kindly provide by Hongtao Liu) and fused with *MtERN1* promoter in pSAT1-cCFP-C (pE3242). *NSP1* and *NSP2* were cloned in to pSAT1-cCFP-C (pE3242) and pSAT1-nVenus-C (pE3228). *MtDELLAs* fused with an *asRed* (a fluorescent protein from *Anemonia sulcata*) tag were cloned by replacing the EYFP in the pSAT4-cEYFP-C1-B vector. NSP2 cDNA was amplified and cloned into pUB-GFP-C-3 × FLAG at the XbaI/KpnI sites. 3 × HA-IPD3 was amplified and inserted into the Gateway donor vector pEntry-topo-SD and then recombined into the pK7GW2-R vectors by LR reactions. Myc-tagged DELLA2 was amplified and inserted into pUB-GFP-c'-3 × FLAG vector, in which 3 × FLAG tag was not expressed, since DELLA2-myc-R primer contains a stop code. For GUS staining assay, promoters were cloned to the vector pEntry-topo-SD. Then they were recombined into pBGWFS7. For complementary experiment, DELLA1/2/3 fused with EGFP (enhanced GFP) at N-terminal was amplified from DELLA1/2/3-pK7WGF2.0, and cloned to the vector pEntry-topo-SD and then were recombined into pK7WG2-R by LR reactions. All primers, restriction enzyme cleavage sites, and short descriptions are listed in Supplementary Table 1 and all the plasmids were sequenced.

### Nod factor treatments

Seven-day-old *della2/della3* double mutants and R108 were incubated in liquid BNM or BNM containing 10^−5^M GA_3_. After 6 h, 10^−9^M Nod factor or H_2_O were added into the medium. After a further 6 h the roots of each group (each group contains 6–10 plants) were harvested and frozen in liquid nitrogen for RNA extraction. AVG was not used in all the media.

### RNA extraction and real-time PCR

Total RNA was isolated with TransZol UP reagent (TransGen) and treated with Ambion DNA Removal Kit. First strand cDNA was synthesized with oligo (dT)_18_ from 1 μg RNA with M-MLV reverse transcriptase (Takara). The qRT-PCR experiments were performed on a BioRad real-time PCR detection system (iQ5) using SYBR Green reagent (Toyobo). The PCR cycle number was determined for each primer pair to the point at which amplification was in the linear phase.

### Yeast two hybrid and yeast three hybrid

The yeast strain AH109 was transformed with the destination vector pGBKT7GW and pGADT7GW containing *IPD3*, *NSP2*, *MtDELLA1*, *MtDELLA2*, *MtDELLA3*, *NSP1*, *CCaMK* or their mutants according to the LiAc transformation method^66^. The expressed proteins were then examined for interaction by dropping 7 μl of yeast suspension on minimal synthetic dropout (SD) agar medium containing the dropout supplement (DO) His-, Leu-, Trp-, Ade- and different concentrations (as stated) of 3-amino-1,2,4-triazole (Clontech). Yeast growth was monitored for up to 7 d. The liquid β-galactosidase assay was performed according to the yeast handbook PT3024-1 (Clontech). The transformation method of Y3H was the same as Y2H, 0.005 mM Met was added into the selection medium.

### BiFC assays

Plasmids were extracted according to the manual of NucleoBond Xtra Midi Plus (Macherey-Nagel). Isolating *Arabidopsis* mesophyll protoplasts and transient gene expression were performed as previously described^67^. YFP fluorescence was recorded by a confocal laser scanning microscope (FluoView FV1000; Olympus).

### Pull-down assays

The coding sequences of *MtDELLA1*, *MtDELLA2* or *MtDELLA3* were cloned into pCold-TF (Takara) for production of HIS-tagged fusion proteins, HIS-MtDELLA1, HIS-MtDELLA2 or HIS-MtDELLA3 in *E. coli*, respectively. The coding sequences of *NSP2* or *IPD3* were cloned into pMAL-C2X (NEB) for production of MBP-tagged fusion proteins, MBP-NSP2 or MBP-IPD3 in *E. coli*, respectively. The HIS-tagged fusion proteins were purified using Ni-NTA Agarose (QIAGEN). The MBP-tagged fusion proteins were purified uing amylose resin (NEB). To test the interaction between NSP2 and MtDELLA1, MtDELLA2 or MtDELLA3, the MBP-NSP2 resin was used to capture the purified HIS-MtDELLA1, His-MtDELLA2 or His-MtDELLA3 protein. To test the interaction between IPD3 and MtDELLA1, MtDELLA2 or MtDELLA3, the MBP-IPD3 resin was used to capture the purified HIS-MtDELLA1, HIS-MtDELLA2 or HIS-MtDELLA3 protein. The monoclonal anti-MBP (Ambmart) or anti-HIS (CW Biotech) antibody was used for western blotting analysis (The information of antibodies in Supplementary Table 2 and Uncropped versions of blots in Supplementary Fig. 8).

### DELLA2 and DELLA3 localization and promoter::GUS analysis

Genomic *MtDELLA2, MtDELLA3* were transferred from the pENTRY vector, described above, into the pK7WGF2 vector by LR reaction. The resulting clone was transformed into *A. rhizogenes* (Arqua1) and for hairy root transformation in *M. truncatula.* Three weeks after hairy root transformation sub-cellular localization was monitored using a confocal Zeiss Axio Imager M1 upright microscope.

Roots of promoter-GUS transgenic plants were used for GUS staining assay. The roots were put into the staining buffer and vacuumed for 10 min then incubated for 0.5 to 3 h at 37 °C. After staining, roots were included in 3% low gelling agarose and sliced into 70 μm sections using a VT 1000S vibratome (Leica). Slices were observed and taken pictures in bright field light microscopy (Nikon SMZ1500).

### *In vitro* phosphorylation

IPD3 was expressed from pET28a in *E*. *coli* strain Rosetta (TransGen Biotech). Expression products were affinity purified via nickel-agarose (Qiagen) under the denaturing conditions by using 8 M urea. Denatured proteins were refolded by stepwise dialysis^26^. Purification of MBP-CCaMK, HIS-IPD3, HIS-MtDELLA1, HIS-MtDELLA2 and HIS-MtDELLA3 was performed according to the protocol of E8200 (New England Biolabs) and of BioSprint96 (Qiagen), respectively. IPD3 was phosphorylated *in vitro* by CCaMK as described^26^. Each reaction was carried out by using 1 μg MBP-CCaMK protein, 0.2 mM CaCl_2_, 0.5 μM Bovine CaM, 2 μg full length and 6 × HIS-IPD3 as substrate. Phosphorylation reactions were performed at 30 °C for 30 min in the presence of DELLA1, DELLA2 and DELLA3. Relative intensities were then calculated for each band by ImageJ.

### Co-immunoprecipitation assay

Co-immunoprecipitation studies of CCaMK/IPD3/DELLA2, IPD3/DELLA2/NSP2 and DELLA2/NSP2/NSP1 were performed. Proteins were expressed in *E*. *coli* strain Rosetta (TransGen Biotech) using pETDuet-1 and RSFDuet-1 vectors. Immunoprecipitation of proteins used anti-S-tag antibody (MBL). Protein A agarose (Merck-Millipore) was used to precipitate the immunoprotein complexes with 50 ml RIPA buffer (150 mM NaCl, 50 mM Tris-HCl, pH 7.5, 1 mM DTT, 1 mM PMSF, 0.1%Triton X-100, 0.1% pellet of cocktail (Roche)) in 50 ml IP buffer. After immunoprecipitation, beads were washed five times with IP buffer. Proteins were then released and collected by boiling in 1 × SDS loading buffer for 5 min. Immunoprecipitation products were detected by immunoblotting with anti-HIS antibody.

For co-immunoprecipitaion in *N. benthamiana*, Flag-tagged NSP2, Myc-tagged DELLA and HA-tagged IPD3 were expressed from *N. Benthamiana* leaves through coinfiltration of *A. tumefaciens* strains GV3101 harbouring proper constructs. Three days after infiltration, *N. benthamiana* leaves were powdered in liquid nitrogen. For each sample, 0.3 g powder was lysed in 600 μl immunoprecipitaion buffer containing 50 mM Tris-HCl (pH 7.5), 150 mM NaCl, 1 mM EDTA, 0.2% Triton X-100, and 1 × EDTA-free protease inhibitor cocktail (Roche). Samples were incubated on ice for 30 min, followed by centrifuge twice at 15,000*g* for 20 min at 4 °C. Partial supernatant (50 μl) was retained for input samples to ensure proteins were normally expressed in all samples. The rest of the supernatant was incubated with a 20 μl slurry of anti-flag M2 agarose beads (sigma) for 2.5 h at 4 °C with a roller shaker. The beads were collected by centrifuge gently and washed once with the immunoprecipitaion buffer. Proteins were eluted by boiling the beads in 60 μl 1 × SDS loading buffer containing 5% β-mercaptoethanol and separated on SDS–PAGE gels. Co-immunoprecipitated proteins were detected by western blot analysis using anti-Flag (sigma), anti-Myc (millipore) or anti-HA (sigma) antibody (the information of antibodies in Supplementary Table 2 and uncropped versions of blots in Supplementary Fig. 8).

### Protoplast transient assay

The protoplast transient assay was performed as previously described^67^. The 2,410 bp *ERN1* promoter was amplified by PCR from A17 genomic DNA and fused with the firefly luciferase gene was used as a transcriptional activity reporter (15 μg of plasmid per transfection). pRNL (a Renilla luciferase reporter plasmid) was used as an internal control to normalize transfection efficiency in protoplast assays (2 μg per transfection). Other effectors of transcription factor constructs were used at 12 μg per transfection. Protoplasts were incubated in WI solution for about 13 h. Protoplasts were harvested by centrifugation at 50*g* for 12 min, resuspended with 200–700 μl reporter lysis buffer (RLB), and vortexed for several seconds. Then they were frozen in liquid nitrogen, thawed at 37°C, centrifuged at 13,500*g* for 1 min and the supernatant (lysis solution) was used for further analysis. Luciferase Assay Reagent (100 μl; Promega) was added to 20 μl of the lysis solution, and luciferase activity was measured with GloMax 20/20 Luminometera.

### Data availability

The authors declare that the data supporting the findings of this study are available within the article and its Supplementary Information files or are available from the corresponding author upon request.

## Additional information

**How to cite this article:** Jin, Y. *et al*. DELLA proteins are common components of symbiotic rhizobial and mycorrhizal signalling pathways. *Nat. Commun.* 7:12433 doi: 10.1038/ncomms12433 (2016).

## Supplementary Material

Supplementary Information Supplementary Figures 1 - 8, Supplementary Tables 1 and 2 and Supplementary Note 1
