# Supplementary material for: DELLA proteins are common components of symbiotic rhizobial and mycorrhizal signalling pathways
Source: Nat Commun. 2016 Aug 12;7:12433. doi: 10.1038/ncomms12433 (PMC4990646; doi:10.1038/ncomms12433)
Supplement: Supplementary Information — Supplementary Figures 1 - 8, Supplementary Tables 1 and 2 and Supplementary Note 1 [file ncomms12433-s1.pdf]

Supplementary Information:

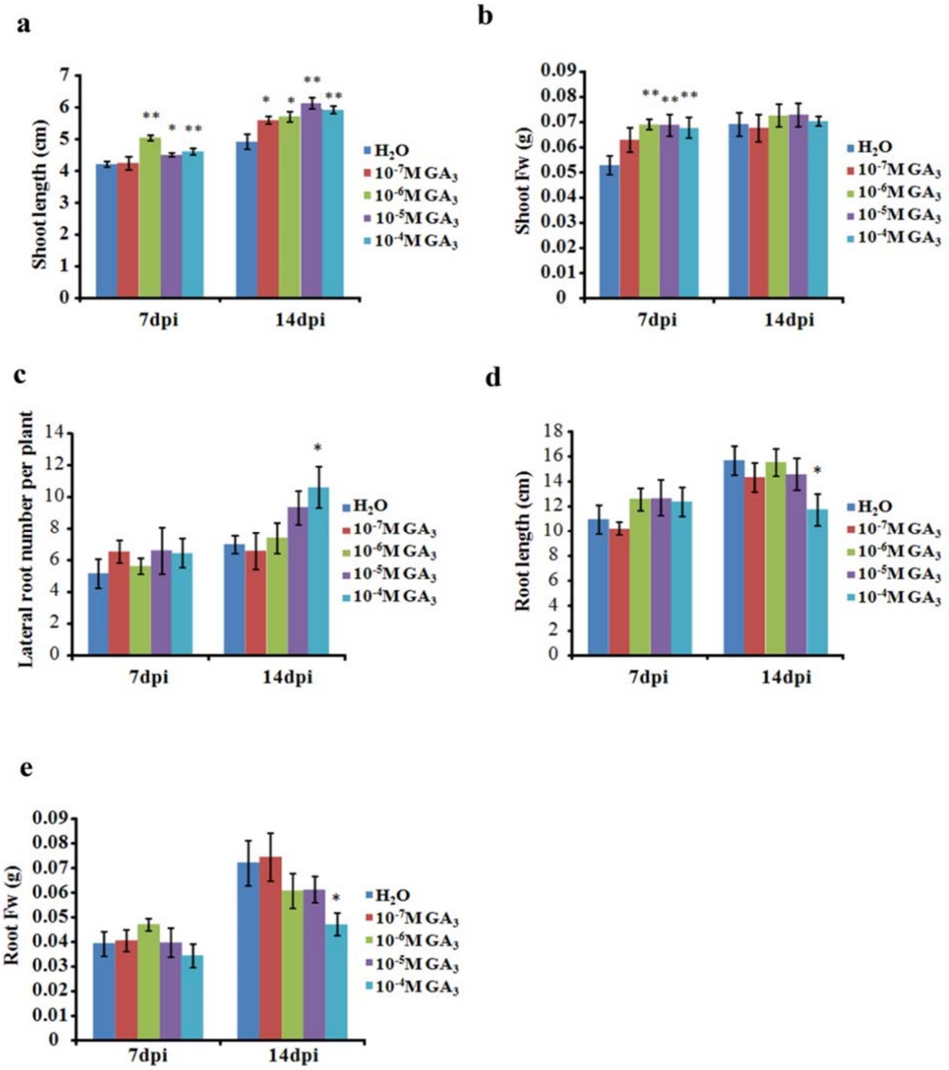

Supplementary Figure 1: Developmental phenotypes of *M. truncatula* plants treated with GA_3 at different concentrations.

Quantification of shoot length (**a**), shoot fresh weight (**b**), lateral root number (**c**), root length (**d**) and root fresh weight (**e**) of *M.truncatula* plants treated with GA_3 at different concentrations. Note that increasing GA_3 concentration promotes the shoot growth but doesn't affect the lateral root number and root growth. 10 plants were analyzed for each GA_3 concentration. Error bars represent standard division. The asterisk indicates a significant decrease relative to control (Student's *t*-test, * $p < 0.05$; ** $p < 0.01$).

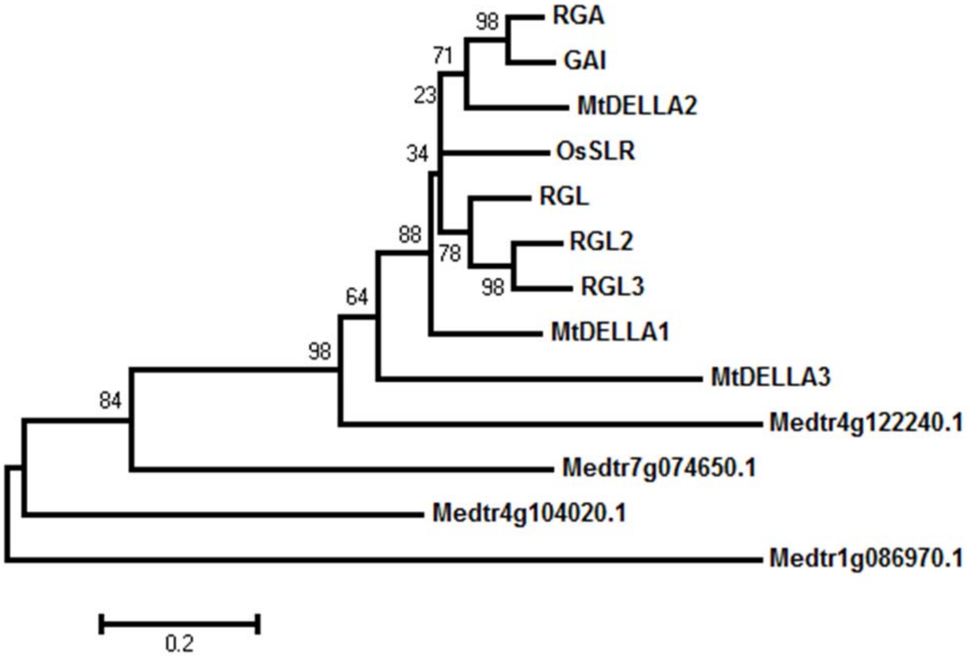

Supplementary Figure 2: Phylogenetic Tree of MtDELLA proteins and the homologs in *Arabidopsis*, *Medicago* and rice (*Oryza sativa*).

The evolutionary history was inferred using the Neighbor-Joining method. The percentage of replicate trees in which the associated taxa clustered together in the bootstrap test (1000 replicates) are shown next to the branches. The evolutionary distances were computed using the Poisson correction method and are in the units of the number of amino acid substitutions per site. Evolutionary analyses were conducted in MEGA6.

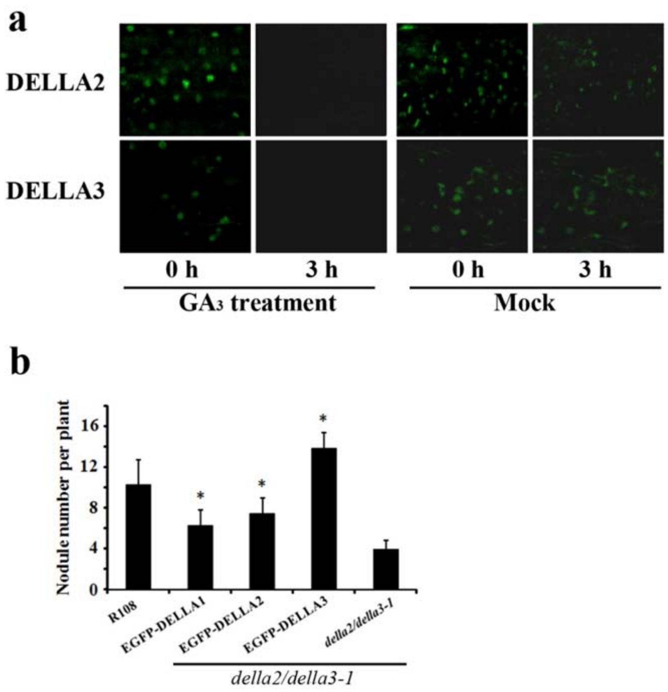

Supplementary Figure 3: Genetic complementation of *della* mutant and GA_3 promoted degradation of MtDELLA2, MtDELLA3 in the nucleus.

(**a**) 3 hours treatment of 10^{-5} M GA_3 caused degradation of MtDELLA2/3. MtDELLA2-GFP and MtDELLA3-GFP fusion proteins of 14-day-old hairy root transgenic plants treated with or without GA_3 (mock) were observed under a confocal microscope. (**b**) *EGFP-DELLA1,2,3* can rescue the phenotype of *della2/della3* double mutant. The roots expressing *MtDELLA1/2/3* were inoculated with *S.meliloti 1021*. the nodules were counted 21 days post inoculation with *Sm1021*. $n \geq 9$, where n denotes the number of plants. Error bars represent standard division. The asterisk indicates a significant increase relative to double mutant transformed with empty vector (Student's *t*-test,* $p < 0.05$).

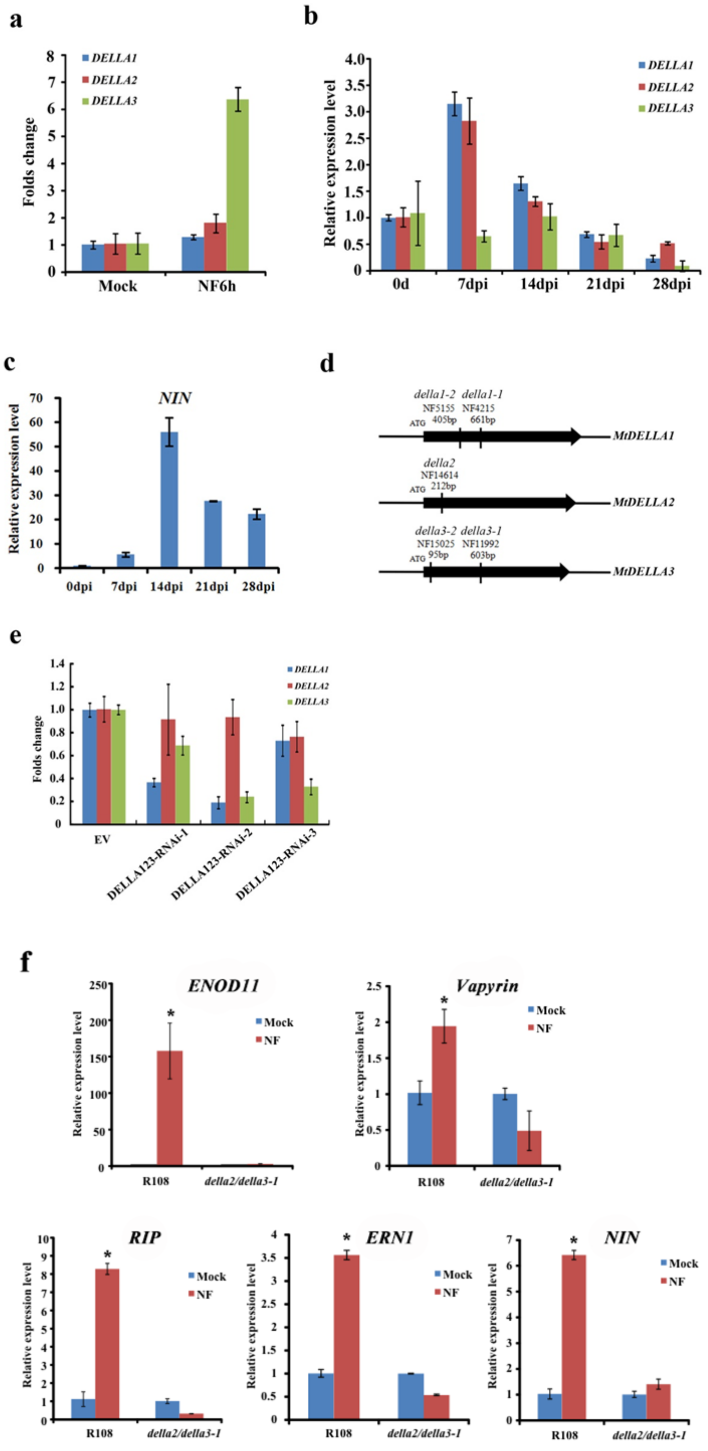

Supplementary Figure 4: Expression of *MtDELLAs* and isolation of the *della* mutants

(a) The relative expression of *MtDELLA1, MtDELLA2* and *MtDELLA3* six hours post Nod factor treatment. Expression levels were normalized against the reference gene *Elongation factor 1-alpha* (*EF1-α*). **(b)** Time course of the relative expression of *MtDELLA1, MtDELLA2* and *MtDELLA3* upon inoculation with *Sm1021*. Note that the expression level of *MtDELLAs* was induced by *Sm1021* at early nodulation time point. (**c**) The expression level of *NIN* was also analyzed as a positive control, *NIN* was strongly induced during nodulation. Fold changes in expression level are shown relative to uninoculated roots at equivalent time points. Expression levels were normalized against the reference gene *Elongation factor 1-alpha* (*EF1-α*). (**d**) The location of the *Tnt1* insertions in *MtDELLA1*, *MtDELLA2* and *MtDELLA3*. Black arrows indicate the coding regions. The vertical bars indicate the *Tnt1* insertion loci. (**e**) The expression level of *MtDELLA* genes in *MtDELLA* RNAi hairy transformed roots. 8 independent transgenic hairy roots were pooled for real-time PCR. Values of relative transcript levels are the mean of three technical replicates. Error bars represent standard division. (**f**) Real-time PCR results revealed that MtDELLAs required for induction of nodulin genes. 7-day-old *della2/della3* double mutants and R108 were treated with 1nM Nod factor or mock (BNM medium) for 6 hours. In wild type the nodulin genes were induced, however these genes could not be induced in *della2/della3* double mutants after Nod factor treatment. This is a replicate of Fig 3c-g.

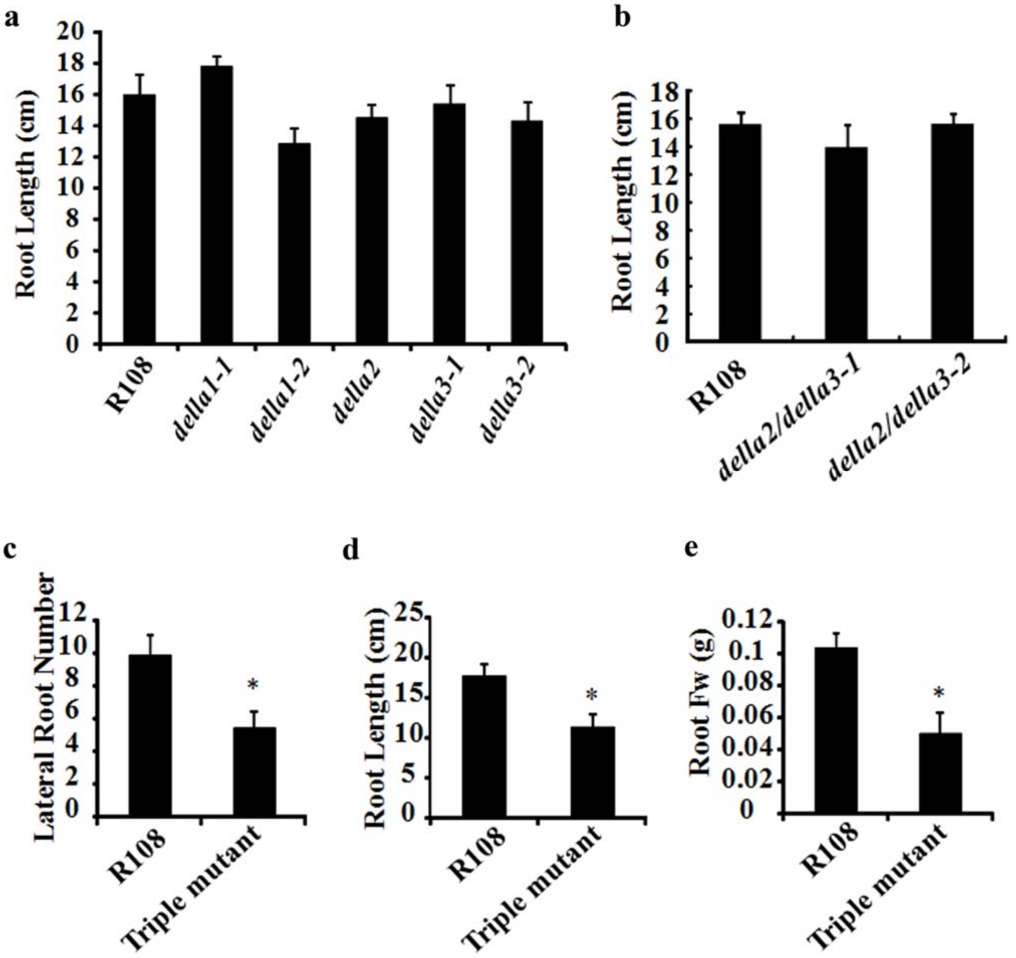

Supplementary Figure 5: Developmental phenotypes of *della* single, double and triple mutants

(**a**) The quantification of root length of *della* single mutants and wild type (R108) at 21 days post inoculation (dpi). n≥8 (**b**) The quantification of root length of *della* double mutants and wild type at 21dpi. n≥8. (c-e) The phenotype of *della* triple mutants. The lateral root number (**c**), root length (**d**), and root fresh weight (Fw) (**e**) of *della* triple mutants were reduced compared to wild type at 21dpi. n≥12, where n denotes the number of plants. Error bars represent standard division. The asterisk indicates a significant decrease with Student's *t*-test ('*' $P < 0.05$).

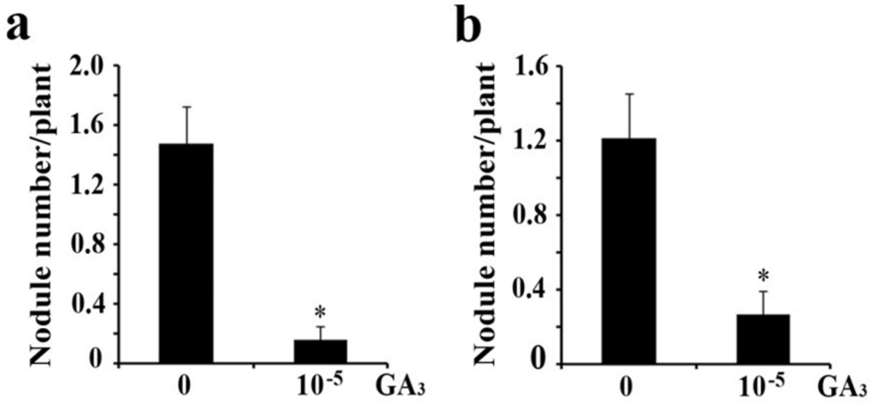

Supplementary Figure 6: The effect of GA_3 on spontaneous nodule formation. (a) and (b) GA_3 inhibits spontaneous nodule formation induced by gain-of-function CRE1 (a) and CCaMK1-311 (b). 20 hairy root transformed plants were assayed. Error bars represent standard error. The asterisk indicates a significant decrease relative to the control with Student's *t*-test ($p < 0.01$). This is a representative experiment that was repeated twice.

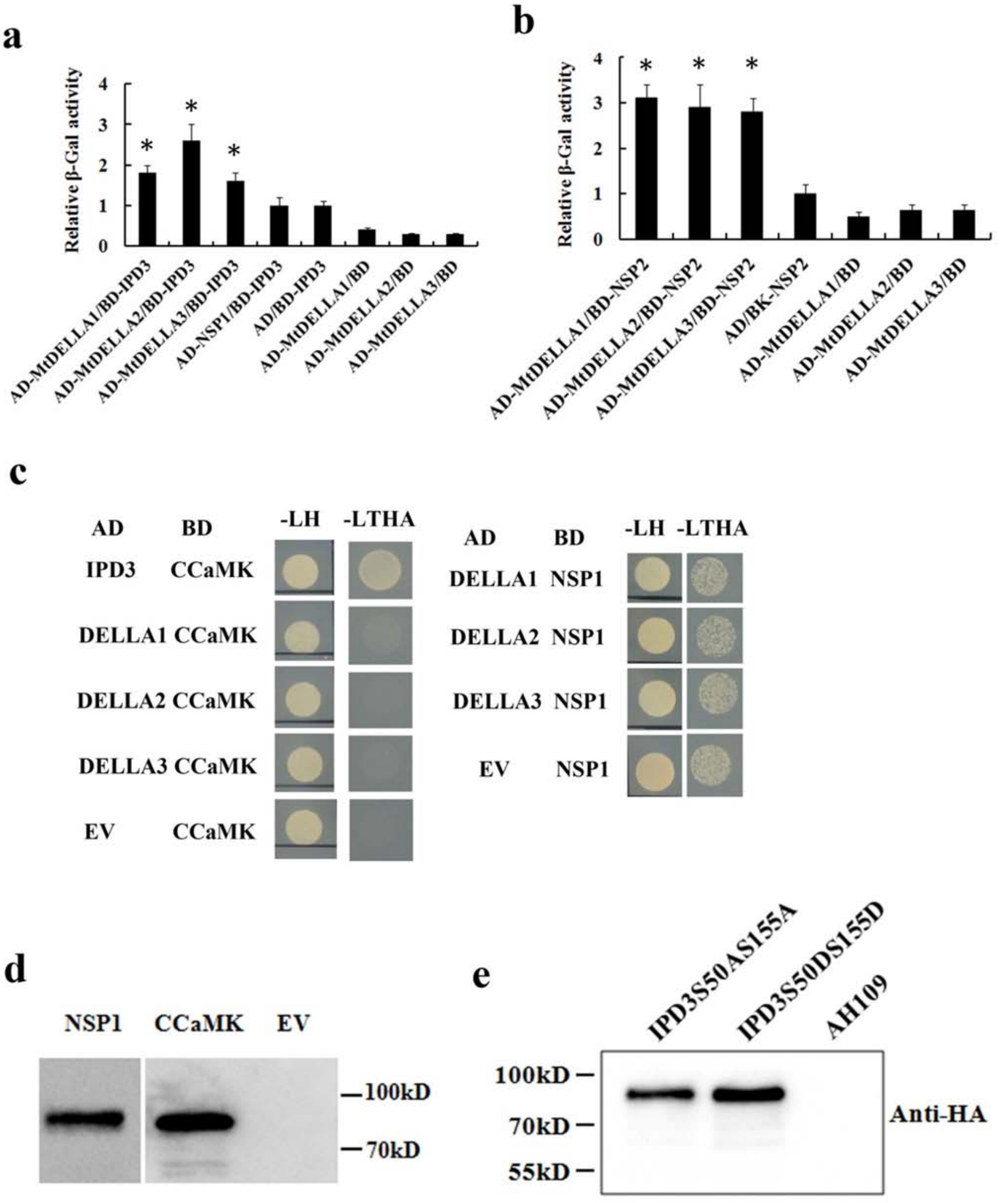

Supplementary Figure 7: MtDELLAs can't interact with CCaMK and NSP1 in yeast two-hybrid assay.

Yeast two-hybrid assay showed MtDELLA proteins could interact with IPD3 (a) and NSP2 (b). Data are presented as relative β-galactosidase activity. Error bars represent standard division. The asterisk indicates a significant decrease with Student's *t*-test ('*' $P < 0.01$). (**c**) No interaction was detected between MtDELLA

and CCaMK/NSP1 in yeast two-hybrid assays. SD/-Leu-Trp-Ade-His (-LTAH) medium with 30 mM 3-amino-1,2,4-triazole (3AT) was used for NSP1 interaction analysis. (**d**) Western blot analysis confirmed the expression of the fusion proteins in *S. cerevisiae*. Recombinant proteins were expressed from pGBKT7 contained a cMyc epitope and were detected by anti-cMyc monoclonal antibody. (**e**) Western blot analysis confirmed the expression of the fusion proteins in *S. cerevisiae*. Recombinant proteins were expressed from pGADT7 contained a HA tag and were detected by anti-HA antibody.

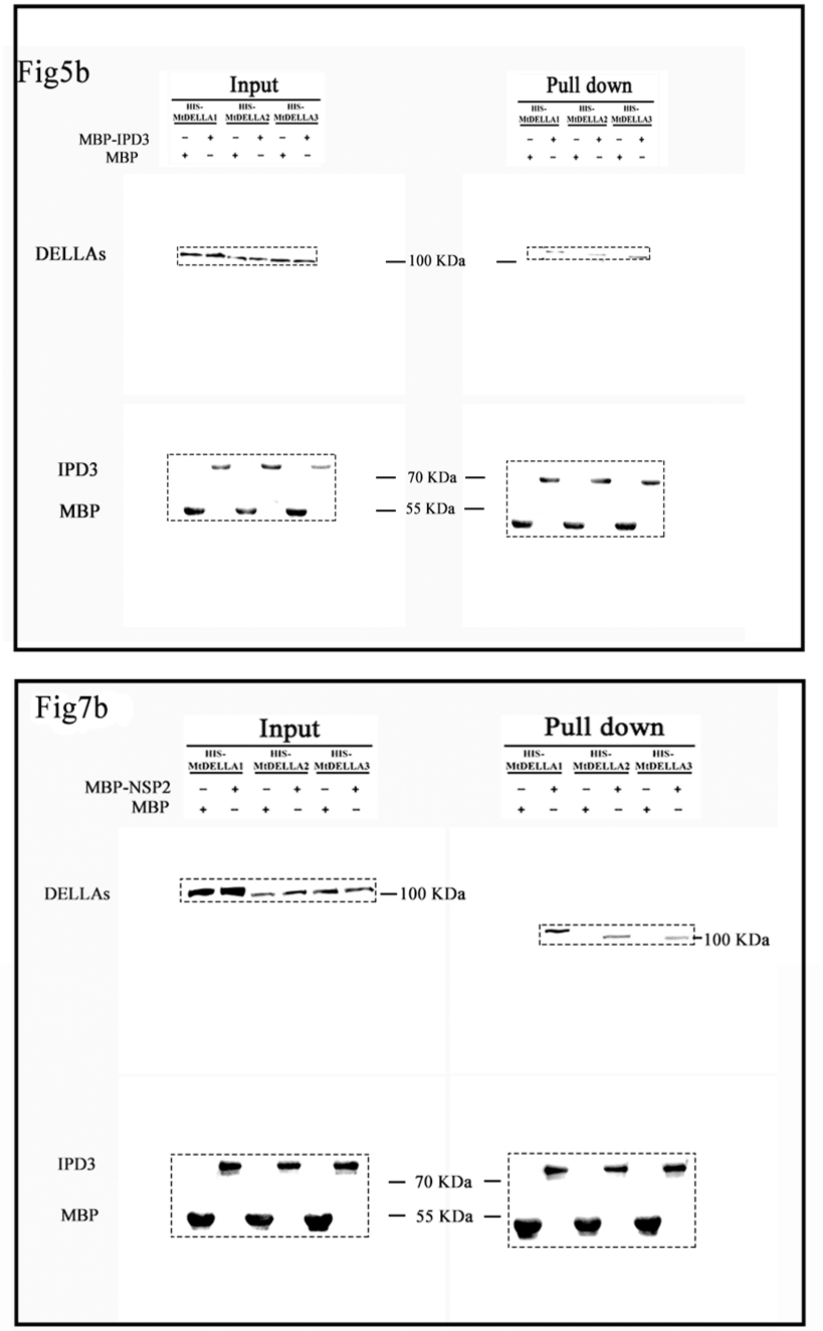

Supplementary Figure 8A: Uncropped images of immunoblotting results in Fig5 and Fig7.

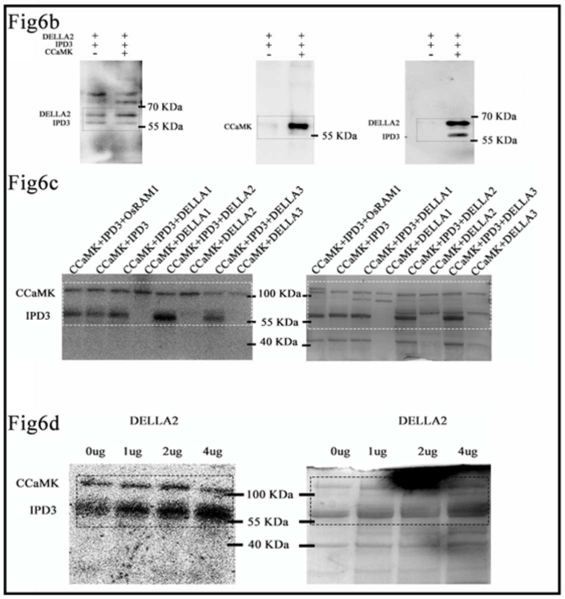

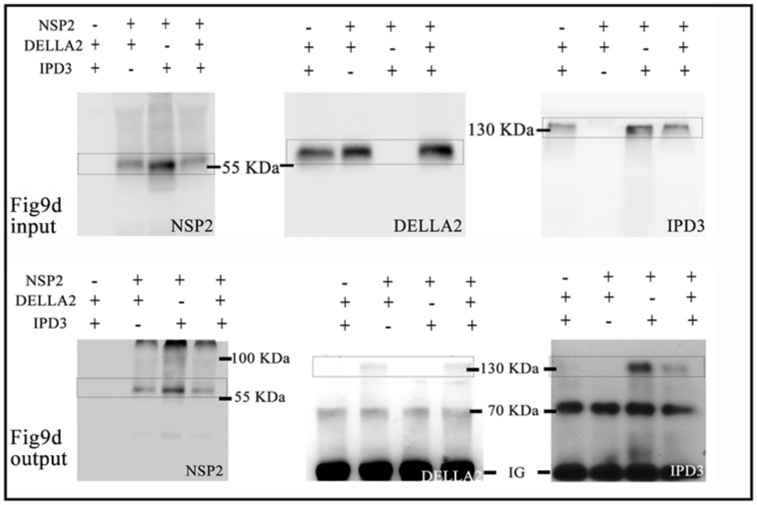

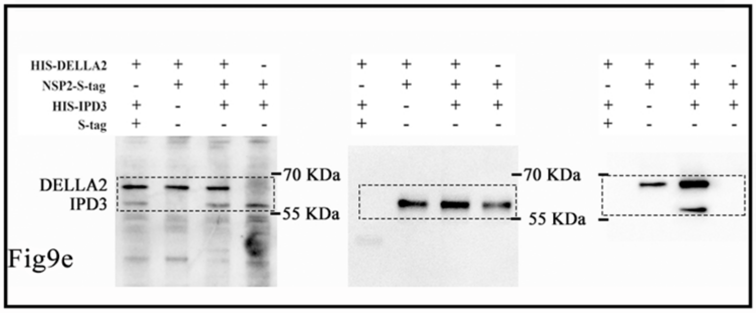

Supplementary Figure 8B: Uncropped images of immunoblotting results in Fig6 and Fig9.

Supplementary Table 1: List of primers used in this study.

Primers used for genotyping	
NF14614-F	CGGAATGCTGTGGAAACACAC
NF14614-R	GTATGGATGAACTCTTAGCTG
NF4215-F	CAACTTTGGTGAATGTTCATCA
NF4215-R	CATAAGCGCAGGCCATTGCATC
NF5155-F	CAACTTTGGTGAATGTTCATCA
NF5155-R	CATAAGCGCAGGCCATTGCATC
NF11992-F	CGGATTCAGATCCGAGAAGACG
NF11992-R	CAATGTCGGCTACAAAGTCCG
NF15025-F	CGGATTCAGATCCGAGAAGACG
NF15025-R	CAATGTCGGCTACAAAGTCCG
Tnt1-R1	TGTAGCACCGAGATACGGTAATTAACAAGA
Primers used in real-time PCR	
NF14614-qF	AGGTGTTTGTTGGGAAGACG
NF14614-qR	GAAAGATGTTGAGCGATGG
NF11992-qF	TGTTGGCAAACATGAACACA
NF11992-qR	AGCTTGATTTGCGGTGAAAT
NF15025-qF	CGGCTACAAAGTCCGATCAT
NF15025-qR	TCCTCTCCGAATTTGACCAC
NF4215-qF	AAGACCGGTGGTTCTTGTTG
NF4215-qR	GCTTGTGCGAAATAGGAAGC
NF5155-qF	GGGTTCAAACCATGCTCACT
NF5155-qR	GCCGGTTCAGATTCTGGTT
DELLA1-qF	CCAACTCGAACTCGACCGGG
DELLA1-qR	CCAGCAAACAAAGCTAACAAAG
DELLA2-qF	AGAATCTACGGTGTGTTTCC
DELLA2-qR	TGCATCCCTTGATTAATCGA
DELLA3-qF	ACGCTCCGTTAACGTTAGAT
DELLA3-qR	CATTTCAATACCCGAATGAT
NIN-qF	GCAATGTGGGGATTTAGAGATT
NIN-qR	GGAAGATTGAGAGGGGAAG
ERN1-qF	GGAAGATGGTGCTGTTGCTT
ERN1-qR	TGTTGGATTGTGAACCTGACTC
FLOT4-qF	CTGAGGCAGTACGCGGACTTG
FLOT4-qR	AGGTGGTAACATTCCCGTTTG
RIP-qF	TAGGGAAAAACGCATTGGAG
RIP-qR	ACAACAGGGCCTTTGCATAC
Vapyrin -qF	TCATCCTCCACAACAACAAGGT

Vapyrin -qR	TCAAGCACTTCTCTTATGTCATCCATTG
MtENDO11-qF	TTCTTGTACTCGCTAGGGTTAGTGTT
MtENDO11-qR	GAGGCTTGTAAGTAGGAGGAGGC
ENOD40-qF	CAATCACTCTATCTATGTAGCACTG
ENOD40-qR	CTCAAAGGAAGACAACACCATC
Primers used in protoplast	
pERN1-AgeI-F	GGACCGGTGTCAAAAGCTACGTTAAGCC
pERN1-BamHI-R	CGGGATCCCACAAAGTTTTTCAAGAAATTG
LUC-BamHI-F	CGGGATCC ATGGAAGACGCCAAAAACAT
LUC-HindIII-R	CCAAGCTT TTACAATTTGGACTTTCCGC
NOS-HindIII-F	CCCAAGCTT GTTTCTTAAGATTGAATCCT
NOS-PstI-R	AACTGCAG CCCGATCTAGTAACATAGAT
NSP1-EcoRI-F	GGAATTCGATGACTATGGAACCAAATCC
NSP1-BamHI-R	CGGGATCCCTACTCTGGTTGTTTATCCA
NSP2-SacI-F	CGAGCTCGCATGGATTTGATGGACATGGATG
NSP2-SalI-R	ACGCGTCGACGCTATAAATCAGAATCTGAAGAAGAACA
DELLA1-NcoI-F	CCCATGGATGAAGAGAGAACACCAAGA
DELLA1-△TAA-SpeI-R	GACTAGTCTTGGACTCATTTTGTGGAA
DELLA2-NcoI-F	CCCATGGATGAAGAGAGAGCATAAGCT
DELLA2-SpeI-R(△TGA)	GACTAGT GTGCGAAACCACCACTGAGT
DELLA3-NcoI-F	CCCATGGATGGAAATAGTTTCAGATTC
DELLA3-SpeI-R(△TGA)	GACTAGT ACAATCAAAACGCAGTGTTT
asRed-SpeI-F	GACTAGTATGGCCTCCTTCCTGAAGAA
asRed-R	TTATCTAGATCCGGTGGATC
Primers used in RNAi	
DELLA1-RNAi-1-F	CACCTTGTCTCAACCCCTTTTCTC
DELLA1-RNAi-1-R	TTCCACCGCCGGCGGTGGTG
DELLA1-RNAi-2-F	CACCTGGTTTCCGTTCTTCCTT
DELLA1-RNAi-2-R	AATCACACCCCCACCAAA
DELLA2-Fused RNAi-1-F	CACCGTTCATTACAATCCCTCTG
DELLA2-Fused	TCAGCACAAGCCATCAAACTATGA

RNAi-1-R	
DELLA3-Fused RNAi-1-F	CGCTTCGGATACGGTCCACTA
DELLA3-Fused RNAi-1-R	ATGTTTGCCAACAACCCTTGC
Primers used in yeast	
CCaMK-SmalI-F	TTTCCCGGGAATGGGATATGGAACAAGAAAACTCT
CCaMK-PstI-R	TTTCTGCAGTTATGGACGAATAGAAGAGAGAACTACA
CCaMKT271D-F	AGTTTTTATGAGAAGGATTGGAAGGGAATTTCA
CCaMKT271D-R	TGAAATTCCCTTCCAATCCTTCTCATAAAAACT
CCaMK-NotI-F	TTTGCGGCCGCAATGGGATATGGAACAAGAAAACTCT
CCaMK-BglII-R	TTTAGATCTTTATGGACGAATAGAAGAGAGAACTACA
DELLA1-NotI-F	TTTGCGGCCGCAATGAAGAGAGAACACCAAGAAAG
DELLA1-BglI-R	TTTAGATCTTCACTTGGACTCATTTTGTGGAAGC
DELLA2-NotI-F	TTTGCGGCCGCAATGAAGAGAGAGCATAAGCTTGAAC
DELLA2-BglI-R	TTTAGATCTTCAGTGCGAAACCACCACTG
DELLA3-F	AAGAGAAAGGTGGCGGCCGCATGGAAATAGTTTCAGATT CTTCTTCT
DELLA3-R	TCGGGCTAATGCGGCCGCTCAACAATCAAAACGCAGTGTT
DELLA1-BamHI-F	TCGGATCCAAATGAAGAGAGAACACCAAGAAAG
DELLA1-PstI-R	TTCTGCAGTCACTTGGACTCATTTTGTGGAAGC
DELLA2-EcoRI-F	TGAATTCATGAAGAGAGAGCATAAGCTTGAAC
DELLA2-BamHI-R	TGGATCCTCAGTGCGAAACCACCACTG
DELLA3-EcoRI-F	TGAATTCATGGAAATAGTTTCAGATTCTTCTTCT
DELLA3-PstI-R	TTTCACTGCAGAACAATCAAAACGCAGTGTT
Nsp2-EcoRI-F	AAGAATTCATGGATTTGATGGACATGGATG
Nsp2-BamHI-R	AAGGATCCCTATAAATCAGAATCTGAAGAAGAACA
IPD3S50D-F：	AAGCTTTCGCACCGATGATGAAGAGCTTTTCAAAC
IPD3S50D-R：	GTTTGAAAAGCTCTTCATCATCGGTGCGAAAGCTT
IPD3S50A-F：	AAGCTTTCGCACCGATGCTGAAGAGCTTTTCAAAC
IPD3S50A-R：	GTTTGAAAAGCTCTTCAGCATCGGTGCGAAAGCTT
IPD3S155D-F：	TACAAGAAGCCGGGATTCTGAATTGCGGGCG
IPD3S155D-R：	CGCCCGCAATTCAGAATCCCGGCTTCTTGTA
IPD3S155A-F：	TACAAGAAGCCGGGCATCTGAATTGCGGGCG
IPD3S155A-R：	CGCCCGCAATTCAGATGCCCGGCTTCTTGTA
IPD3-NotI-F	TTTGCGGCCGCAATGGAAGGGAGAGGATTTTCTG
IPD3-BglII-R	TTTAGATCTTCAAATCTTTCCAGTTTCTGATAGAA
CCaMK-BamHI-F	AAGGATCCATGGGATATGGAACAAGAAAACTC
CCaMK-SacI-R	AAGAGCTCTCAGGCTTTCTCACCTTTGACC

Primers used in BIFC	
DELLA1-SacI-F	AAGAGCTCAATGAAGAGAGAACACCAAGAAAG
DELLA1-KpnI-R	TTGGTACCACTTGGACTCATTTTGTGGAAGC
DELLA2-SacI-F	AAGAGCTCAATGAAGAGAGAGCATAAGCTTGAAC
DELLA2-KpnI-R	TTGGTACCAGTGCGAAACCACCACTG
DELLA3-SacI-F	AAGAGCTCAATGGAAATAGTTTCAGATTCTTCTTCT
DELLA3-KpnI-R	TTGGTACCAACAATCAAAACGCAGTGTT
IPD3-SacI-F	AAGAGCTCAATGGAAGGGAGAGGATTTTCTG
IPD3-KpnI-R	AAGGATCCAAATCTTTCCAGTTTCTGATAGAA
NSP2-SacI-F	AAGAGCTCAATGGATTTGATGGACATGGATG
NSP2-KpnI-R	AAGGATCCATAAATCAGAATCTGAAGAAGAACA
Primers used in vivo/vitro experiments	
CCaMK-XbaI-F	AATCTAGAATGGGATATGGAACAAGAAAACTC
CCaMK-PstI-R	AAACTGCAGTTATGGACGAATAGAAGAGAGAACTAC
IPD3-BamHI-F	TTGGATCCATGGAAGGGAGAGGATTTTCTG
IPD3-SalI-R	TTTGTCGACTCAAATCTTTCCAGTTTCTGATAGAAT
OsRAM1-EcoRI-F	AAGAATTCATGGCCGGCGGCGGCGCGAAGCTCC
OsRAM1-BamHI-R	AAGGATCCTCAGCACCGCCACGCGGAGGCGGCG
DELLA1-BamHI-F	TCGGATCCATGAAGAGAGAACACCAAGAAAG
DELLA1-SacI-R	TTGAGCTCTCACTTGGACTCATTTTGTGGAAGC
DELLA2-BamHI-F	TCGGATCCATGAAGAGAGAGCATAAGCTTGAAC
DELLA2-SacI-R	TTGAGCTCTCAGTGCGAAACCACCACTG
DELLA3-EcoRI-F	AAGAATTCATGGAAATAGTTTCAGATTCTTCTTCT
DELLA3-SalI-R	AAAGTCGACTCAACAATCAAAACGCAGTGTT
CCaMK-BglII-F	TTTAGATCTAATGGGATATGGAACAAGAAAACTCT
CCaMK-KpnI-R	TTTTCTCGAGTTATGGACGAATAGAAGAGAGAACTAC
NSP2-F	GCTGACGTCGGTACCATGGATTTGATGGACATGGATG
NSP2-R	AGACTCGAGGGTACCCTATAAATCAGAATCTGAAGAAGAACAAG
NSP2-Flag-XbaI-F	GCTCTAGAACCATGGATTTGATGGACATGGATG
NSP2-Flag-KpnI-R	GGGGTACCTAAATCAGAATCTGAAGAAG
IPD3-HA-BamHI-F	TTGGATCCATGTACCCATACGATGTTCCAGATTACGCTTACCCATACGATGTTCCAGATTACGCTTACCCATACGATGTTCCAGATTACGCTATGGAAGGGAGAGGATTTTCTGG
IPD3-HA-SacI-R	TTGAGCTCTCAAATCTTTCCAGTTTCTGATAGAAT
DELLA2-myc-F	CTACTAGTGGATCCGGTACCATGGAGGAGCAGAAGCTGATCTCAGAGGAGGACCTGAAGAGAGAGCATAAGCTTGAAC
DELLA2-myc-R	TGGTCCTTATAGTCGGTACCGTCAGTGCGAAACCACCACTG

Primers used for promoter activity assay	
pDELLA1-F	CACCGAGTATGCCAAAACACGATGAC
pDELLA1-R	GTTGATTAAATTGGTGAAGTG
pDELLA2-F	CACCGAGTATGCAGTCATAAAGTAC
pDELLA2-R	CTTTCACTTTTTCAGTTTTCTC
pDELLA3-F	CACCCGTAATGCCTGAATCTCTACG
pDELLA3-R	AGAATTCAAAACTTAAAACACAG
pNSP2-F	CGGGATCCGTCGACAAGCTTTCCGTACATGTCACC
pNSP2-R	CCGGAATTCGGTATAATTAAGTTAGGTGT

Supplementary Table2: Sources and working concentrations of antibodies used in this study.

ANTIGEN	ANTIBODY	DILUTION	SOURCE
HIS-MtDELLA1/2/3	anti-HIS tag Mouse Monoclonal Antibody	1:2000	CW Biotech, CW0083
MBP-NSP2/IPD3	MBP-Tag (6d3) Mouse mAb	1:1000	Ambmart, M40003
CCAMK-S-tag	anti-S-tag pAb	1:2000	MBL,PM021
Flag-NSP2	ANTI-FLAG® antibody, Rat monoclonal	1:4000	Sigma, SAB4200071
Myc-DELLA	Anti-Myc Tag Antibody	1:4000	Millipore, 16-213
HA-IPD3	Monoclonal Anti-HA antibody produced in mouse	1:4000	Sigma, H9658

Supplementary Note 1.

Yellow indicates CYC-box

Green indicates NRE-box

It worthy to note that CYC-box (TGCCATGTGGCA) only found in promoter of NIN and half of CYC-box (TGGCA) was found in promoter of other nodulin genes.

> The promoter of Lj*NIN*

TTTGTGGAGATGCATTGCGTCTATGAATTCACCAATGAAACAGAGGAGAATCTTTTTTTC
AATTTTCACGGCCTCAACTTGGAATGTTAAAATATATTATCAAATCTTTTTCACAATTGTTG
CCATCCACTTGTACTGGTAATTATATAGTATTAATTATCATACGGCAATAATGTATGTAAT
TGTATGTGTAATGTAGATGGTAGAGACATCTATTTCGCCGATATCGTAGACATGTACGCAA
ATTATATAAGTTTGCATTTTTAGGTACACAAATTTTGTACGATTGCCATGTGGCACGCAGA
GAGGAGCCCACAAGAGGCGAGACCCTATTTCTCTGTCTCTCTTTAATTAATTTTGTTTAGA
GTCAAGTTCATCATGATAATCTGCCACTATTATATAGCCGGTGTTGCGCATAGATGGGTAG
ATATAGATATATGTTATTAATTACAGCTGCTACAGCCACAGAGTACAAAGAAAGAATTGA
AAGAAAGGCGTGAGACTTTACATTACATTATTAATTGATGATATAGAAATAGAAATAGAA
ATAGGAATAGGTAGAGTCGACAGACATCCCGAATTACTAGCATTTTGTACCCTTCAATCCT
ATAATAACAGGAATAATATACCATTCTTGTCTCTTTCTGAATTTACTCCCTTATTAATATTC
TTTCCTCAAGTCATGAGTAGTTAAGTTTTAGAAGCTTGTTGTTTTGCTGTGTTGTTTCTTAC
TCGAGAGGCAGCAAGCATATACTGAATACTACATTTATTTATAGAATATAATAATAATTTA
CAGCTGATAACTATGAAAGAATATTTATTTATATGTTATATGTATTGTATATACCTGCCAC
GGTGAGGGGGGATTAGGGATATAAAAACTGGAGACCACTCTTTGATTTTGCTTACACTTGT
GGGTCCTAGCAAGATACAATTAAAGAAATTTTGGGTTTTAAATAAGAGACTTGTTGATCG
CATGTTGAGGCAAGGCAATCATTTT

>The promoter of *MtERN1*

GTCAAAAGCTACGTTAAGCCATGCAATTATTGTACAACTTACATGATTAACGAACTAATAT
AAAATTATGTCGAAACGAATTTTATTAAAAAAATTAATACATGATTATTGCAATATAGGTT
GATTTAGTCGATAAAGAATTGAATCATAGCTTATAATGACGTGAATTCAACTTAATGACTC
TCGAGTCCCCTTGTAATCCAACTCACCCAAATTTTGTATACCATAAAAATAAATTAGTATA
TGGTTATTGCTAACATAAATATAAATATGCTTGAATCTCGAAAAGATTTTGAAAGATAACA
TTACTGTGTTGGATTGCTAATTTAATTTGATATAATAAAGTTGTGTCTTTTTCCCCATATCT
TCTTTCTTGTCTAACGTGAGATCCATTGTGAACTTTAGTGGCATGCAATATTTTAGCAAATT
GTACTATATGCAAATCAATAAACAAAGAAGATAATGACAATGTCACACAAATCAAAAGCT
TGGAACTGGTTGAAGAAGAATCTATTAATAAACACAATAATGATCAAATACGGAACAAAG
TGTGTCGTTGGTGACGTGAGAAGAGGGCTACAACTCAAAACAAACGTAGCTAGCACACAC
ATGTAGGAATCTTTATTATTACATGTCTATCACTTTTCCATTGCAAATATGTATAGAAATA
GATATATTTCTGGGTTTTCTTCTTTAAGATTCAAACAAAGTATTCAATGGCAGTAATTATTAA
TTTGTAAAAAAAAAAAAGACAATAATTATTAATTAAATATAAAATACTTGGCAATTAAAA
AAATTTATAACATATCCAACTTTTATTTTTTCTAAGAAGATCAATGATAACAAAAAATCA
ACAATTAAAAAGATCCAAATGATATGGTAGGGTGTTTGAATGCAAAAGAAGAAAAAGAA
CAAAACAGACAATTTATTGAAGAATGAATGATTAATATATTTTCCGTAGAAGAGAGCGAA

TTATCTCTTTTTAATATTGTTTGGACTAAATAGTTCTTTAATTTTATTTTTTTTGAAGTAAAA
TAGTTCTTTAATTATTCATAGGGACGTAATTGTTCTTTATCTTTTCGTGATTCAAGTATTCC
AATTTCCTTTTTCAGGACTAAATGTTGGAGTAATTTTTTTGGGTCCAAATTAGTCCTACGAA
TTTTCCTTAGGGCTTAAATTGATCTTGACTATTATTATGATTATTATTATTATTATTTATTAT
TAGTTTTTTAAATAATATTGACATATGGTTGAGGACTTGAGGTGGCAATGACCTGTTTCAT
CTTAGTGCTAAGCATTGCTTTAGTCTCTTTGTTTATGGGTGCTTGCTAGGATCATTTTGGAG
CCTCCAAGTGGCAGTTATTTGTGCCTTCACCATAATAATTTCAACTGTTTGACTTAACAAA
AAGAGGGGAAGAAAGGGGACAAGAACTAGGGTACCCTAAATTCTACCTAACCTTAATGT
ATTTTCTTGTTTTTCTAAGTGATTGGTGATTCCTTATTCTTTAATCTTTAGCGTGGCTTTAAG
ATCATTTTATTTGTAGGGTTGCTGGTATTCCACAATAATTTCAAATTTATCCAACCAAATAT
TGCCCATTGGATTTAGAAGAGAAGATCTGAATTTGACAAATTTGTTCTATATTATTGTAAA
ATTTGCTTTTAAAATATTTTTTTATGTAGTCTATTGAGTTCGTTCCGTAATATTGACTTGAA
ATTCTGAAGTGTGTTTTTCTTCAAGATCTCATGTTCGATTATCTTTGGTGTTAATATGGGTG
TGTTAATTTAACTTCTTCAAAAAATAAATTGATGTAATTCATTATGATAACATTTATTAATT
TTGTCTTTAAATTATCAATTGGAGATTCTATTATTCAAATTAAAAAAATTATTTGATGCAAT
TTACACATGAGAATTTTTCAAAAATAAAACATAACAAAAATACTATGCCATTAATTTATTA
GTTAGGGACAGTATATTGTTAGAATTTAGAATGTAGGTCTTTAGTTACATAATAATATGCT
TTTGAGAAGAAATAAAAAGATTAAAAATAAACTAGTTGATTTTGTTTTTGGCACATACAA
CCAAATTCCAAGCTTGTCTGCTCCCAAACGACAGCTATAGGAGTGGTCATTCTGTTGAAAA
ATTAAGGGCATCACCAATTGAATTTCAAAGGTACCTTGTCATTATCATACCAAAAAATTAT
ACTTATAATCCCAAGACAATGTGCATGTAATATATTAATCCAATTTGCTTGCTGAAAAGCT
TAACAACTTTTTCACACACATATATATAAAGTCTCATAGCTTGCAAATTACAACATGAAAA
ATTAAGTAATAGCTAGCCTTCCATTATATCTTCATATCACAACATCTTTTCCACTCAAATAC
AATTTCTTGAAAAAACTTTGTG

> The promoter of *MtEnod11*

TTTCACACAGGAAACAGCTATGACCATGATTACGCCAAGCTTGGTACCGAGCTCGGATCC
ACTAGTAACGGCCGCCAGTGTGCTGGAATTCGCCCTTGAGCTCAATATGTTCTTCAGGTTT
CTACTTTCTACCCCAGGCCAACGAGGCATGTTAAGATCCTTCGCATATCTGATTTCTTTTAA
ATCTAGACACGATAATCTACGTGAGTTGCTTTCGGGATTTGAAAGATCTTTTTTGAAAGGT
TTGAGAACCATAAAATTAAGGAGGTAAAGTGCTCGTGGTTGGGACACGTAATTGTCTTGG
TATTAGATCTTTCTTTATTGATATCACTTGCTAAAAGTATATAAGCAAATGGAGGAAAACC
ACTACATGGGGACAGCCCAATTTATTTACGGAGGTCCAATGATTTTTTAGCAAGTGTGATG
TTAGAACTTTTTAAAACCATGATAACATTTTTTTTTTTTTGAGGAAAAAAAACATAATAA
CATTTTATTAAAAGGTATAATATTTTTATTTACATCTTCAAATTCTTAGAAATTTTTACATT
TTCTAAATCTTGTTATTATTGTGAATGTAAATTTGTATCTGAGTAATGCAATCATACGTTTG
AACTTCTATGATTTAAGGATATTTTTCATCTTAAGTTTTTTTGTCAAGTTTTTCTTGTTATGT
TTAAATAAATAATAAAAAATTAGACACTTAAATTTGAGGGTCATAATAACATAAAATAAT
TGCAGGCCTAAAGCTATTATTTTACCAGCCTTTTAATTTGACCTGCCATGGCCAATAAAAC
AAACGGGTCTTTCTTGAAAAAACTGTTTTATAGTTTGGCCTGCCATAGCCAAGAATCTAGA
TTTCCTAATTTTCCTAAAACATTCTAAAAAGTAAGAAGTTTTTACATTTTTGAAAATTATTC
CACAACAGTTTTTGCTTGTTGATAATAATATTCCCTACTAACTTTTTGACATTTAGTGGAGT

GATTGTAGAGAGATAAAAAAAAAAAAAAAAGTTTTGTATTAAGACCTTACACTCCCATTGA
GGTCCCAAACTAGTATAAAACCTAAGCATTCCCTATATGTTATGCACACTAAAATCACTAC
CTAAA

> The promoter of *MtNIN*

AAAAGTAGGTTAGACTAAACAATATTTTACAAGGTGTATACTTGGTTTGATAAAATAAAA
AAATAAAAAAAATACAAGGTCCTTTTAATAAAAATGGTGAAAGTCTTTTTGAAAACTTTAT
TATGGTATGTATCCCAACTTAAGTGTTTATTATTTATTTTGGTTAATTTAAATGTTTATTTTT
TATTTTTTTTTGTCAATTAGTCTAGTGGTTAGACTCACACACATTATGTGTGGAGAAGTGG
AGAATCTGAGGTTCGAACTCCGGTCCTTGCACAAATTATCTCTGGTAGCTACCAACTGAGC
TACACTTACGAGACAATTTAAATGCTTATTTAACATTATTGTTTTTAAATAATTAATCCATT
TAATGACCAACGAGCTAATAAGTCAATGAAATAAAGTTATATTTTGATATTAGCATGGTA
ATCAAAAAGTATGACTTTGTATAACAAACTATTTCAATTTTATTTAATAATAATAATAATA
ATAATATATTCAAATATGCCAAAAAATTACTGTGACCTAATATGCTTAAATTAATAAAAAT
ATTTTCACTAAATAAGTTGAAAATGTTTAACAAGGTATAATGTATAATATAATAAGAATA
ATTATAAAAAACAAATTATTCATAGTCGGTCTAATAATTTTAAAAGAATTTTTTTGTGGTC
TGAAATCTGATATTTGTTCTTAACTAAATAGAGTTGTCTTTTTTTTTTTTTAATATAGTTTGA
GTCTTGTCTATTTAACAAAAAAAAAAAAAAAGTTTGGCTTGCGCTTGAAGTAGGCTCAGAC
ATAAGACTATGTTGATTTGATTTAACCTTCTTCCATCCCTAACCACGATTGTGTCTTCACAA
ATATTAAAAACGTTATTTATTGTTCTTGTGAGCTTATCACAATTGATAGGAACAACGCATA
ATATATGCAAAGTCCGAGGTACGAACCCCAGACACCAACGGCGAAAAAAGAAAAAAAAA
TCTTATTTATCGATTGTTCAACATCGTAATTAAATTCAAAAGAGCTTATTTTTTTATTTGAT
TACTTATTCAAAAAGTGATTTTGCATATGATTTGTTTTTAATTTTGAACACAATTACTTTTT
TAATCAATATCTACATCTAACTATTTTACATCTATTGTAATTAACAAGAAACACTAGCTAT
GATGAGAACATAAACTAATATAAATTATCAACAAATTTAATTTTACAAAGTTTTTTCAATT
TGATATTATATTTAAATGTGAAAGGAAGTAATAGGTAGTTAGGGAGACAGAGATCCCAAA
GTTACTAGTATGATTTTGTACCCTGCCAACTATTAATTGAATTCAACGGCAAGAATAATAT
ACCTATCCTTGTCTCTTTCTGTCTCTCATTGATTGAGTTCATATAGTACTAGTACATGTTGA
TAGTATATATGTTTCTATGCATGTGTTGCTCTCAGCACGAGTGTATGTTATGTTATGTTATG
TTATACAAGAAGCAGCAAAAACACATATAGAAGAAGGGAGTATAATATAATAAAATAAA
ATAAAATAATTTACAGCGCTGCTAATTGTGAAAGAGAATATAGAGATATAAAATATAGAT
ATAGGCCAGTCGCTGCTTTAGCTTACTAGTGGGTCCAACTAAACTAAAAATATAAAGAAA
TTGAAATTGCATTTTTGGTCATTAAATAGGAGACCTGTTGATCATCTAAATATATCATATC
AATTAAGCAAAAGCATTGACGCAAGGCTCTGCCTGCACAAAATACTACTTCTTCTTAATCA
TTCATTCACTCACTACAACTAATTAGGTTAATCTCTATCTATCCTGCCTGCAGCTTCACACA
CATCACATACCTAATTTATTTCCTTGTTTCATTTCTCTTTAATTTCCGTACTTATTATTCTAC
GTACGTGTTCTCCTCAACTACTTAACTTAATTTAATAATTGACTCTTCCTTCATTCCCTTTCT
CTAATATATATCATGACATGAATGATATCTTCTGATCTTTTTATATAGGAGGATCTGAGAA
ACAACTTTAATTATAAGG

> The promoter of *MtDELLA1* 2030bp

GAGTATGCCAAAACACGATGACCAAAAGTGTAATTAAGCCATATATTTAATAGATGCCAT
TAATTTTGAGATATTACATATGTTTCGAACTAAGCCGAGACCAGACACAAGACAAACTTC
AGCTAGGGAAGGTACACACCAAGGTGCTACATCATTTGACCAAAAAAGAGCGCTACACCG
CTAATAATACTAGCGTACTTTAACCCACTTAGACTGGTTTGGTAGTATTGATTTGAGACTT
GTGAGTGTACTTCTTCTCAATGTTACAAATTCGATTTTCCCCGGAGTCAATTTGGACGGAT
TAATTTAGTTTTTTCAAAAAAAAAAAATACTAGCATACTTTTCCATCAATCAATCACCACA
TGATCTACATCACGCAAAAATCGATTTGTAGATCACACAAAAATATTTCAACGACTTCAAT
GAAATTTACTTTGATTATCTTAAATTCCCGCTTATAAAATGTTAGTTGCAACATAAGAAAG
ATAGATTTCTATTTTAATAAAAATCTCACTCTACTCAATCATCAATATTTGTCCAAACAAC
ACTTTTTACTCTATATCGTACCGTTGAACATCGATGACCACCTCAACGAACTCCAAATCAA
ATTTTTATATCCAAAAACATATTATTGTTGATTTCAACCTTAATTTCTAAATTTCATTCTAA
TTTCTCATCCTAATACGATTTTTTTAGGGGTAAAGTGATTGCTGTGGTTACTAGTACAACA
CTAATGCTAATTGTACTAATCTCAACGGTAAAGCCCCAAACTAAAGAGAAATGATATTTG
TACAACTACTTTGTGATAATTTTTAGACAACTTTTTCTCTCATACTTACATTATATTTTTACC
CTCTCTCTTTCTTTTTCTCTCTCCATTATTTTTGACCAATGAAAAGAGAGAAAAAGAAGATT
TTCAAATAGGTTGTACCAAATAATTGTTCAAATATCATTCCTCCAAACCAAATGTAAAAAG
TGTGAGCAAGAATTGGAAACCACGCGCTATCAGAACGCGCGTGAATGGGCCAGGACAGA
GGTGGAGCCCATGATCATTCTCGGTCCCCAAGACCCTCAATTGATGAAATGTACGGCCAC
CTGATATTTCTAGTGTTGTATGGTTTCTGTGCTATGCACAAGCTGTACACTATCATGGGCAT
CTTTTCTTCCTTCACATTACGGTACTGTCACGCCCCTCTCTCTCCTTTTAAATTAAGCATAT
GTTTGCTTTGTGTTAATTTGTTTTAAATAAGTTAATTTAATATTTATGAGCTTAACTCAATT
GATAGAGACAATGTATAATATTTATAAGGTTGAATTTCAAACTCCAACCACAAAAAAAAA
GATTGATTTAGATCAAAAGCTGTTTAAAATGAGTTGGAGATAAAGTAATTTAATATGTAA
AGTTGAGTATTTAAATCAATTATAAAATTAAAAACCTTAATTTTTAAACTTTTTTGAGTCA
ATTAATTTTAACAACAAAATCAAAGGAATCAAATGTATTAGAATCAATTCTAAATTCTTAA
AATCAATTTTGATCTTTTAAAAGTTAAATCAAATATACGCTAATCAAAAGAAAATGAGAT
GAATATTAATAACCAATATCGTAATAACCAAAAAAAGTTATACACTAAACCTTTTCCTTTT
TCATTCTTCATTTTCCTTTTATTTACACCACCACGCAAAATTTACTCACCCCCACACTCTAC
TACTAAAATTTCCCTAATGTAAAACTTTTATCTAATTAATTTCTCTCCCCACAATTATCAAC
AACCTTTTTATTTTAGCAAAAAAGCATACACCAAAAACAGAAAAGAAACTAGTTGAAAAA
AATTCCATTATTATTATGGTTTCCGTTCTTCCTTATCTTTTTCATTTTCAATCTCCTCTCTCTC
TCTTCCTCCTTTCTCTTGTCTCAACCCCTTTTCTCCCATTCTTCTAAACTTCAAACCATAAAC
TCAAAAAACCCCATTTTGCTATGACCCAACTCAAATTCTAAATCAAAATCTATTCACTTCA
CCAATTTAATCAAC

>The promoter of *MtDELLA2* 1121bp
GAGTATGCAGTCATAAAGTACATAAGTCTTTGATAATTGTGTGTCCATAAAGTAAATTCAT
AGGTTAATATCTGCAGAAGGAAGAAGAAACAAGGCTTAAGTTTGTGAATTTGCAACTTAA
TTAATGATATAATTTTTATAATGCAGGAAATGTCATATCTATTAGTTGCAGCATGTTAAAT
AGATTATATCATAGACCTTCTGCATGAAGTCAGTGGATCACATTGTACTACAAAGTTATGT
GATATATATCAATTTTCTACTACTATGACATTCTTATCTTGATTTATTTCAACAGCTTGATT
TTTAATGTTTGATTTTTAGTTTTACCTTCTTTGTAAAAAATTTATCTTGCTCCTTGTTGATAA

ATATGAATGAAAAGGCTGCACATGACAAAACCATGATTTATTTGGCATATATTCGTTCATT
TTCTTGTGAATAGAATTTTTATGACAATAGGTACAATATACTACTTGAAGAACTAAATATA
TGATTTTTGGTAAGAAAAAAATCTAAATTTTTAAGTTTAGGGAACATTTTGCACATAAGTG
CAAAATTTCGGGGGTATTTGATCATAACCGTAGACAGTTAACAGAAAAATTTGACGGAAG
GATAAATTGATTAGCGTTATACAATTTTAAAGAGATAATTAAAATTTTGTTAATTTCAAAG
ATAATTGACGAAACTTACAATTTTAGATAGAAAATTGACTATTTACTCTCACAAAAGTCCA
ATTAAGCCTACAAAATAAAGAGAGTTTATGGCTCGTCGTCTGACAAAAAAGCAAGAAATG
GAAACAAAGTAAAAAACATAAATGTATATTTTGGCGGATCTTATCTGTTACTGTTAGAACC
CTGCCAGCCCGGCCTTTTGATTGGGTCTCGAGCCTGCCACGAGCGTTTTCTACTCTCTTTCC
TTTTTTTATTTTAAATATTTTATTTTAATTTATTATAAAAATAAAAAAAAGATTTGGATATA
TATGAGTTTTACGGGTATATGATTGTGTTGATGAAAGAAAGAAGGTTAGAACTTAGAAAC
ACCAGTGAAAAAGAGTCACAATCAGAAAACAAAACATAGAAAGTGTATTGTGTGTGTGTG
AGAGAAAACTGAAAAAGTGAAAG

>The promoter of *MtDELLA3* 2431bp
CGTAATGCCTGAATCTCTACGTCTTTGTGAGAACGATGGAGGGTATAGATAATTGGTTTCT
TTGTTTAAATTTGGTTGAATTGGTCCTTCTCAATTTCATGATAAGAAATGTTCTTATATAGT
GCCAAGGCAGGCAGGGTGAAAAAATTATTTAAGTAGCTAAGTTGTGAATATCGCAATTAA
ATTAGTATTTAAATCAACTTTTGTCGTGATTACACTAATAAACACATTATTTATTGTGCAAT
ATGTAACTTCATATGAATCATAATGGGTGCTATAGCTTTTATATTGATTAAACATAAATTA
TACCTTAAGATGCTTTGTGTGACACATGGTTACTACAGGAATATTCTAGTTACAATGACGT
TCTACTTGAAGGAAAGACTGTACTTTTAGGTACCTATAATAATGCAAATTAAAATAAGGG
GAATTAATCGCTTTAGTCCCTAAATGGGAAAGGACTAATCATTTTAGTCTCTGAATGTAGA
CTCTGTTGGTCAATTTTAGAGACTAAAGTGACTAACGGAGTTTACATTCATAGACTAAATT
GACTAACGGAATCTGCATTCAGGGATTAAATTGACTAACAGAGTCTACATTTAGGGATTA
TTTTATAATTTATTTGCATTCAGAGACTAAAGTGACGACTCATTTCCTATTCAAGGACTAA
AGTGACTAATCTCCTTTAAAATATTTTTTTTTATTAAGAGTAAGTAGGTACGTAATTAAGC
AAGTAGGGTCTTAGCAGAGACAAAAATCAAAGGGCCACTTGTTGCAATAGAAACATAAA
CAGGAAAAAGCTACCTACAATTAAGTTGTAGAAAATTTGATCAGACTAAATCAACCTTTA
CAAATGATTATAGGAACATAGGCTAAGATAAGACAAGCATATACAAAGAGAGATACACA
AAGTGACTTTACACAACAGCATCTTAGAACTTTACAAGCTTTTAATATGATGAGACTAATT
GGGAGACTTTGTACTTTGGATAATGACAACTTAACATTGAGTTCATTACACTCAAAAAAG
GTTTTCAAATTTGGGCAAGTGTTATATTTTAACCAATTTACCACTTAACTTGAAATCCTAAT
TATTTGAGGCAAAAGATAGTGACAGTGGGACTAATATTTTCACCAAAACAGTTAAGCATT
CTCTAATAAGTGAATCGGATGGAATGATCACAATAAATAGATAATGTAGAAAGTAGAAAA
TTTAAATATGACCCATCATCAACCTCTCTGCCTCATTGGGTCGGGTATTGATAATTACATG
TCCAAAAACTATAAAGCACGGATACGGACATGGACATCGGACAGGATACAGACACTGAC
ACGTCGATACAACACAACTAATAATTTGATAAAATCACATAATTCAGTGTAATTACAAGT
GTCAGTGTGGTATCGGTATCGGACACGACACGTGTCCGACACTGCGACATGTCTAACCCT
AGAAGTGTCCGTGCTTCATAATCCAAAAATATAATGTTACATGAGTTGATTTGATGTAAGC
TAAATTTCTCAATGTGTAGGCTATGAAGTACAGGTTATCTGTAGTATACCGTGGGTGCTAA
ATGAAGAAAGTAAGAAAAACAAGCTCGTGAAAGCAAGGTGCTGTCAAGTGTGTCAGGGT

TTTTTCTCATCTATGAATTGAATACAAATTATCACTTGCTAGGTTTAGCAGGTATAAGATA
GGAAATAAATTCAAAAACAAATTGAAGAGTAGCATAACATAGCATGTGAAATAACAGTA
TTCCCAAGTTGGAATTGGAGTAAAATTGGAATCAACATGTTAGCACATTCCTGTTTATATA
TTATTTGGCATTTGTTTGGTCAGTTAAACTGTAAACAACAGTAATACTTTACACTGTTTATA
TATTTTTACAGTTTGTGGTATTATTGTACAGTTGTACTATAGTTTCTGGACCCTGAAAATCG
TAACAAATCACAATGGTTAATGGTGGTAGTGTGGTACTACACTGATAGTAGTAGACCCAG
ACCGACCCAGGAGGAACCACTTGTTGTACCCAAATCCCAATATTGGATAAACAGCATCTA
AATTATCAAAAGCCAACTTTGGTACCTAAGGCTACTAAAAATTGAAGGCACACTAGCGTG
ACATTAGGGTACCTTTTAAAAAAAGTTGAAATTATATAAGTAGTTGTATCAATCAAATATG
ATGTGCTAGCACTGCAGATAGCTACTCAACACAAATTAGTAACCCATCGTGCTCATGCATT
TTACAACTTAGCTTCTCTCTTAGTTTTCTCACACATCACTTTCTTTATTTCTTTCTCCTCTTTT
TTCACTCTCTCCTAGCTTAAACTCAATTATAAACACACACACTTCTTTAGATGCTATCATAC
TTCAACTTTGCTTCCACAGACTAACGCTTATAAGTTCAAAAACTGTGTTTTAAGTTTTGAAT
TCT

>The promoter of *MtIPD3* 1047bp
AAGTGGAGTCAAAAGAAATAGTTTATATGAAATAATAATATCACAAGTCACCTACACATA
TAAATATTTGTATAAAGTGATGGAACACACATAAATTTCATTAGATAAAAAAACGAATAA
CACTTGTACGTAACTAATAATTTATACGACATGACTAGACTTTAATGTTTTACATAACAGA
ATAAATTTTCACAGTCCTATAAAGAATTTATGGACGTGAGGTGAACAAACATTGCTGAAA
CATGCTTAGCAAACAATGAAAGCACCACCTTAACAGCTTCCTATTTTAATACAAAAAACA
ACCAATCAATGTTCCACAATATCCCTTTCATAACACCATCATCTAATTGCATCACCTACGG
CATTTTATTTATTTAATAATATCAAAAGGTGACCAAAATTGTCATTAAATTAATCAAAATG
ACCCTCTTTGCTTGATTCAAAAGCTACAAATTTTTTTCATTTTTAAACCTGTCCATTAAATT
TCCACACACGGTTTTCTAACCTTTGAAGATTAATTTTTAACATCACAATCTTTTCTCTTTCA
TTGTACACAAGAGACAAATGAATGGTACATGGAATCTTTTGAGTATTTTTTTCACTCTTAG
ATGTCATAGCCACTGCTCTAATATTTAGTATTTATTAATTCTATTGACAAAAACAAAAATC
AGAAAAATATTTACTATTAGTAAATGCCAAGTTCTAAGACAAAGTTTATTTATCTATATGC
AAGATTTCTTTCAAGTTTCACGTGTAAATTGTTGTAGGAAGCTATTCCTTTAACTGTTTCAT
GTTAATTAGTTACTACATGCTTTTGGAATAAAACAGTTCATAAAGTCTTTCTTTCATTTCCT
TGGTTTTTGAGAAGAAAAAATAGTTGCTAGCTTAGGTTGAATTTTCATTGAGTATTCAAAA
TTCTCTCCCTTGGTTTTTGAGAAGGGTATTGTGATGAATAAAGAATTCAGCTGAAAATTCA
TTTATGAAACCTGAAAGATCTTAGCCAAAAACCTGTGTTGAAAATAAGTTCAAGCATCATT
CAAGTGTTTC

>The promoter of *MtNSP2* 1463bp
AAGCTTTCCGTACATGTCACCTATAAAAGGTCAATGACCTCTAGTGTAGGTAGGTTCTCAG
TTGTGATTAAACTTCTAGAATCGCCTAAAAGTCTCACTGACTTGACTGTTCGAGTCCTTAT
AGGTACACAACTCTCTCGTGGATTACTAACGCCATCTTAAACCGGTTCTAACCAACTCTCG
GTTTCGTCTTCAGGTCCACTCTAAATCACTGACACCGTCTGTAGGAATCATTTTATAATATT
TTTCATCATTCTGAACATTTCGATGGCTTGAAACAAGAGAGCAGCCACTTCAAAGACGAG
GACTTGTGATCATGCTCAAAAGTATTTAATTAAGAAGGAAAATGCTACTTTCAGCGAAAC

TTTTTTTCGAATACTTCACGACTTGCAAATTATAACTGGAGTAGGTCCTCTTTCTTTTCACTTCCACTTCCATGTTTTCCGCTTAATAGAATATCAACGATCCTAGTTGATTTGCAATCAATTCGTGAGAATATTTCTGGCTGCAAGTAGCAGCACTCAATTAAAAATTAAATATTAGAAACAAAAATAAAAAAATAACATCTACTCTTATACCGTCTAGCCATCAGTCATATGGGTGAAAAAAATATATACAACTCAATTTGAAGACTCGTTAAGTGCTAATCTCATTTTCCTCAAAAAAAAAAAAGTGCTAATCTCATTTACTTGCAAATTAACCATCAAATTTGTCCGATCTGAAATCATATTGGAGCATAAAGAATTGATCTGATCAACATTGAAAACGAGTTATAGTTGTGTATAATATAACACTCAACTTGTCCATTACTCACCCTTGATTATTAGTTCCATTATGTGATGAGACTTAAATAATAAAAAAAAAAAACAATATTAATTAACAACTCTAAATATACAGACATTGATTATTTGAGAAGTCAAATGGATAAACATCAAACCAAACTATGAGCTGGGTGAGATAGATGGAAACTTGAGGCTCGTAAAAAGTTATGTATAAACTCAAGGGGCTTATGAGCAATTGAAAAAAAATTACATAGGTATAGTGACTATGCTAATCTATAAAATTAAAAAAAAAAAAAATCAAGAGCAAGACAAAGTTGGAGTATAAATAAAAAACGGCAGTCATAATTATTTACGAATATACTCTTTTATAAGTTATTTATTTCATAAAATATTTAAAAATAATTTGCATTCACATCAGGTAGGAGGTCATGTTGTTAGTGACAACAGCGCACATAACAATATTCCCTTAACTTACCAATCAGTTTTTGGACTTCCACTGTCTCATCCTCTGAATCCTTCTTCTCTCTCTCCATCACCTCTCAACCCCTTTATAAACCTCTTCCCATACCATTCATTCTCCCTCACCACACAACAAACCACAACTCAATAATAATATACACAAACCTAACTATATATTATATTTTGCTGATCTCTTTCTCTTAAACACCTAACTTAATTATACC
